# Unique Footprint in the *scl1.3* Locus Affects Adhesion and Biofilm Formation of the Invasive M3-Type Group A *Streptococcus*

**DOI:** 10.3389/fcimb.2016.00090

**Published:** 2016-08-31

**Authors:** Beth A. Bachert, Soo J. Choi, Paul R. LaSala, Tiffany I. Harper, Dudley H. McNitt, Dylan T. Boehm, Clayton C. Caswell, Pawel Ciborowski, Douglas R. Keene, Anthony R. Flores, James M. Musser, Flavia Squeglia, Daniela Marasco, Rita Berisio, Slawomir Lukomski

**Affiliations:** ^1^Department of Microbiology, Immunology, and Cell Biology, West Virginia UniversityMorgantown, WV, USA; ^2^Department of Pathology, West Virginia UniversityMorgantown, WV, USA; ^3^Department of Pharmacology and Experimental Neuroscience, University of Nebraska Medical CenterOmaha, NE, USA; ^4^Shriners Hospital for ChildrenPortland, OR, USA; ^5^Section of Infectious Diseases, Department of Pediatrics, Baylor College of Medicine, Texas Children's HospitalHouston, TX, USA; ^6^Department of Pathology and Genomic Medicine, Center for Molecular and Translational Human Infectious Diseases Research, Houston Methodist Research Institute and Hospital SystemHouston, TX, USA; ^7^Institute of Biostructures and Bioimaging, National Research CouncilNaples, Italy; ^8^Department of Pharmacy, University of Naples Frederico IINaples, Italy

**Keywords:** biofilm, M3-type streptococci, *Streptococcus pyogenes*, Scl1, Scl2, ECM, colonization

## Abstract

The streptococcal collagen-like proteins 1 and 2 (Scl1 and Scl2) are major surface adhesins that are ubiquitous among group A *Streptococcus* (GAS). Invasive M3-type strains, however, have evolved two unique conserved features in the *scl1* locus: (i) an IS1548 element insertion in the *scl1* promoter region and (ii) a nonsense mutation within the *scl1* coding sequence. The *scl1* transcript is drastically reduced in M3-type GAS, contrasting with a high transcription level of *scl1* allele in invasive M1-type GAS. This leads to a lack of Scl1 expression in M3 strains. In contrast, while *scl2* transcription and Scl2 production are elevated in M3 strains, M1 GAS lack Scl2 surface expression. M3-type strains were shown to have reduced biofilm formation on inanimate surfaces coated with cellular fibronectin and laminin, and in human skin equivalents. Repair of the nonsense mutation and restoration of Scl1 expression on M3-GAS cells, restores biofilm formation on cellular fibronectin and laminin coatings. Inactivation of *scl1* in biofilm-capable M28 and M41 strains results in larger skin lesions in a mouse model, indicating that lack of Scl1 adhesin promotes bacterial spread over localized infection. These studies suggest the uniquely evolved *scl1* locus in the M3-type strains, which prevents surface expression of the major Scl1 adhesin, contributed to the emergence of the invasive M3-type strains. Furthermore these studies provide insight into the molecular mechanisms mediating colonization, biofilm formation, and pathogenesis of group A streptococci.

## Introduction

Group A *Streptococcus* (GAS) or *Streptococcus pyogenes* is a human-specific Gram-positive pathogen responsible for significant morbidity and mortality worldwide (Carapetis et al., 2005; Sims Sanyahumbi et al., 2016). The clinical outcomes resulting from GAS infection range from superficial pharyngitis and impetigo to severe life-threatening diseases, such as streptococcal toxic shock syndrome and necrotizing fasciitis, as well as post-infectious sequelae, including rheumatic fever, rheumatic heart disease, and post-streptococcal glomerulonephritis (Cunningham, 2000). Historically, GAS has been a significant cause of puerperal sepsis, scarlet fever, erysipelas, and pharyngitis (Stevens and Kaplan, 2000). GAS strains are epidemiologically subtyped based on nucleotide sequence variation at the 5′-end of the *emm* gene, reflecting differences in the hypervariable N-terminal region of the anti-phagocytic surface protein M. Over 240 M-types have been identified (http://www.cdc.gov/abcs/index.html), and certain M-types have been shown to have nonrandom associations with specific disease outcomes (Cunningham, 2000; Shulman et al., 2004). Since the 1980's there has been resurgence in invasive GAS diseases in the U.S. and other parts of the world. Numerous epidemiology studies conducted in the U.S. (Stevens et al., 1989; Musser et al., 1991; Cleary et al., 1992; Johnson et al., 1992; DiPersio et al., 1996; Cockerill et al., 1997), Canada (Davies et al., 1996; Kaul et al., 1997; Sharkawy et al., 2002; Hollm-Delgado et al., 2005), and Europe (Gaworzewska and Colman, 1988; Colman et al., 1993; Lamagni et al., 2008; Meisal et al., 2010) have found associations between infections with M1- and M3-type strains and invasive diseases. Specifically, M3-type strains have been associated with severe invasive disease (Musser et al., 1991; Lamagni et al., 2008) and fatal outcomes (Gaworzewska and Colman, 1988; Colman et al., 1993; Sharkawy et al., 2002).

These epidemiological observations have spurred significant whole genome sequencing efforts aimed at identification of the underlying genetic basis for virulence in M3-type GAS. The complete annotated genomes of invasive M3 strains MGAS315 and SSI-1 have been reported (Beres et al., 2002; Nakagawa et al., 2003). These studies have revealed that differing phage elements, insertion sequences, and the large-scale chromosomal inversion identified in SSI-1, contributed to much of the genetic variation between M3 and other M-types. Acquisition of prophages and SNP's drive the expansion of different M3 subclones during epidemic waves of infection (Beres et al., 2004, 2010). Strains causing pharyngitis and those causing invasive infections are derived from the same pool of M3 strains (Shea et al., 2011). In addition, genetic variation in virulence regulators, including RopB and CovRS, which are “hot-spots” for the accumulation of mutations, also contributes to the hypervirulence of M3 strains (Beres et al., 2010; Carroll et al., 2011; Shea et al., 2011; Olsen et al., 2012; Flores et al., 2013; Cao et al., 2014). The cause of the hyper-invasive phenotype of M3 strains is multifactorial and involves multiple virulence factors controlled by complex regulatory networks that continually undergo remodeling.

The streptococcal collagen-like proteins 1 and 2, or Scl1 and Scl2 (also known as SclA and SclB), are major GAS surface adhesins known to contribute to pathogenesis. Both Scl proteins contain an N-terminal variable region, followed by a collagen-like region containing Gly-X-Y repeats and a cell-wall-anchored C-terminal region (Lukomski et al., 2000, 2001; Rasmussen et al., 2000; Rasmussen and Björck, 2001; Whatmore, 2001). Transcription of *scl1* is positively regulated by the multiple gene regulator Mga (Rasmussen et al., 2000; Lukomski et al., 2001; Almengor and McIver, 2004). Scl1 binds cellular fibronectin and laminin (Caswell et al., 2010), and contributes to the formation of biofilm by strains of multiple M-types (Oliver-Kozup et al., 2011, 2013). GAS adherence and biofilm formation is enhanced on extracellular matrix (ECM) coatings and on fibroblast-deposited ECM network (Lembke et al., 2006; Oliver-Kozup et al., 2013), supporting a role for Scl1 in tissue-microcolony formation described during GAS skin infection (Akiyama et al., 2003). While the *scl1* gene has been found in every GAS strain tested (Lukomski et al., 2000; Rasmussen et al., 2000), the *scl1.3*-allele in M3-type strains harbors a null mutation within the coding sequence (Lukomski et al., 2000). A rare natural reversion of this polymorphism was identified in a small subset of M3 carrier strains (Flores et al., 2015).

Scl2-protein expression is regulated during translation by varying numbers of short CAAAA repeats downstream of the start codon that determine whether the protein-coding sequence is in-frame or translation will be prematurely terminated (Lukomski et al., 2001; Rasmussen and Björck, 2001); genome sequencing indicates majority of the M3-type strain contain in-frame *scl2.3* alleles (Beres et al., 2004; Meisal et al., 2010). Scl2 has been shown in some strains to bind the human thrombin-activatable fibrinolysis inhibitor (Påhlman et al., 2007) and contribute to adherence to fibroblasts (Rasmussen and Björck, 2001), although its role in GAS pathogenesis is less understood. Recently, the crystal structure of the Scl2.3 globular domain, which is structurally conserved between Scl1 and Scl2, has been reported (Squeglia et al., 2013, 2014) providing insight into the potential binding interactions with host ligands.

In this study, we show that invasive M3-type GAS harbor two unique conserved features of the *scl1* locus including the IS1548 insertion in the promoter region and the null mutation in the coding sequence, which results in a secreted instead of cell-attached Scl1 protein. We demonstrate significantly different expression patterns of *scl1* and *scl2* in M3-type GAS compared to representative strains of M1-, M28-, and M41-types. We demonstrate that the expression of the Scl1 adhesin is deficient in serotype M3 strains, as opposed to M1-, M28-, and M41-type strains. However, the Scl2 protein is upregulated in M3 strains compared to M1, M28, and M41 GAS. The M3 strains lacked significant biofilms on cellular fibronectin and laminin coatings, compared to M41-type GAS, and did not form tissue microcolonies in a wounded pseudo-organ skin equivalent model of infection. Recombinant Scl1.3 specifically bound cellular fibronectin and laminin, and restoration of surface expression of Scl1.3 conferred significant biofilm formation by M3 strains. Inactivation of Scl1 expression in biofilm-capable M28- and M41-type GAS resulted in larger skin lesions produced by the mutants in a mouse model of subcutaneous infection, supporting a role for Scl1 in maintaining a localized infection. Our model advocates that the lack of surface-associated Scl1 adhesin in M3-type strains causes decreased tissue adherence and decreased capacity for stable microcolony formation, thus, promoting bacterial spread over localized nidus of infection.

## Materials and methods

### Bacterial strains and growth

MGAS315 and MGAS10870 are fully sequenced invasive M3-type strains (Beres et al., 2002, 2010). MGAS315 was isolated from a patient with streptococcal toxic syndrome in the 1980's (Musser et al., 1991) and MGAS10870 was isolated from a patient with soft tissue infection in Ontario in 2002 (Beres et al., 2010). Additional strains from epidemiologically diverse M-types were used for comparison: MGAS6183 (M41), MGAS5005 (M1), and MGAS6143 (M28). The MGAS6143Δ*scl1*-, MGAS6183Δ*scl1*-, and MGAS10870Δ*scl1*-inactivated mutant strains have been previously described (Han et al., 2006a; Caswell et al., 2007; Flores et al., 2015). Group A *Streptococcus* cultures were grown in Todd-Hewitt broth (Becton Dickinson and Co.) supplemented with 0.2% yeast extract (THY medium) and on Brain Heart Infusion agar (Becton Dickinson and Co.) at 37°C in an atmosphere with 5% CO_2_. For antibiotic selection, erythromycin (5 μg mL^−1^), chloramphenicol (10 μg mL^−1^), kanamycin (200 μg mL^−1^), and spectinomycin (100 μg mL^−1^) were added to the medium.

Cloning experiments were performed in XL-1 Blue and TB1 *E. coli* cells, while protein expression experiments were performed in BL21 and TB1 *E. coli* cells grown in Luria-Bertani media (Difco Laboratories) at 37°C. For antibiotic selection, chloramphenicol (10 μg mL^−1^), kanamycin (50 μg mL^−1^), spectinomycin (100 μg mL^−1^), and ampicillin (100 μg mL^−1^) were added to the medium.

### PCR assays

#### Analytical PCR

##### Detection of IS element upstream of scl1 in GAS strains

The presence of IS1548 upstream of *scl1* was determined by PCR with primers IS1548F and Scl1R using Qiagen Taq DNA polymerase (Qiagen, Germantown, MD) as follows: 95°C, 5 min-[95°C 1 min, 62°C 1 min, 72°C 1 min] × 30 cycles- 72°C, 10 min. Sequences of primers used in all PCR assays are listed in Table S1.

##### PCR amplification of *scl1.3* and *scl2.3* alleles

PCR was performed on genomic DNA isolated from M3 strains with primer pairs 232 Up/232 Rev for *scl1.3* amplification and sequencing, and Scl2.3 F/R and SclUp/SclRev for length polymorphism analysis and sequencing of *scl2.3*, respectively. Amplification was performed using Qiagen Taq DNA polymerase as follows: *scl1.3*: 95°C, 5 min-[95°C 1 min, 55°C 1 min, 72°C 1 min] x30 cycles- 72°C, 10 min; *scl2.3*: 95°C, 5 min-[95°C 1 min, 51°C 1 min, 72°C 1 min 45 s] × 30 cycles- 72°C, 10 min. All PCR products were analyzed in a 1% agarose gel with 1 kb Plus DNA ladder as a molecular size marker (Life Technologies, Grand Island, NY).

#### Quantitative reverse transcription PCR (qRT-PCR)

##### RNA isolation

Total RNA was isolated from the logarithmic phase (OD_600_ 0.5) GAS cultures using RNeasy Protect Bacteria Mini kit (Qiagen), employing an additional pretreatment step with 250 μg lysozyme, 100 μg proteinase K, and 12.5 U mutanolysin per sample to augment cell wall disruption. Trace genomic DNA was removed by incubation with TurboDNase enzyme (Ambion). RNA quality was assessed in 1% agarose gel and spectrophotometrically; A260/280 and A260/230 ratios of >1.8 were considered acceptable. RNA was used immediately in cDNA synthesis or stored at −80°C for no more than 1 week.

##### Determination of transcription by qRT-PCR

Synthesis of cDNA was performed using iScript Select cDNA synthesis kit (Bio-Rad) with 1 μg of RNA per reaction and random primer mix. For each sample, a no reverse transcriptase control was performed, containing only RNA, reaction buffer, and random primer mix, to ensure the absence of genomic DNA. Incubations were carried out on a Bio-Rad C1000 Touch Thermal cycler: 25°C 5 min, 42°C 30 min, 85°C 5 min, and cDNA was stored at −20°C until used in qRT-PCR. qRT-PCR reactions were performed with 250 nM primers using SsoAdvanced SYBR Green Universal Supermix (Bio-Rad). For each qRT-PCR reaction, template cDNA, diluted 1:20, control genomic DNA, or no reverse transcriptase control were used. PCR was performed using the following conditions on a Bio-Rad CFX96 Thermal cycler: 95°C 2 min- [95°C 5 s, 60°C 30 s] × 30- [65°C − 95°C, 5 s, 0.5°C/step]. Standard curves were generated for each primer set using cDNA from MGAS315 to determine the linear range and estimate reaction efficiency. Gene expression of *scl1, scl2, emm*, and *mga* was normalized against the expression of *tufA* gene, which has previously been identified and validated as an appropriate endogenous control (Virtaneva et al., 2005). The ΔΔCt method was utilized to compare gene expression between MGAS315 and other M3 strains, as well as M1, M28, and M41-type strains. Data was averaged from three independent experiments, each containing three technical replicates. Statistical significance was determined using an unpaired *t*-test.

### Recombinant Scl (rScl) proteins

#### Cloning, expression, and purification of M3-derived rScl proteins

The rScl proteins were generated using the *Strep*-tag II cloning, expression, and purification system (IBA-GmbH, Gottingen, Germany). Proteins were expressed with a C-terminal affinity tag and purified on *Strep*-Tactin sepharose, as described (Han et al., 2006b). rScl proteins rScl1.3V and rScl1.3FL are derived from MGAS315, whereas rScl2.3 protein is derived from the M3 strain MGAS3375. The recombinant Scl2.3V protein has been described previously (Squeglia et al., 2013). Construct containing the recombinant full-length Scl1.3FL (rScl1.3FL) protein was previously described (Flores et al., 2015). Briefly, the *scl1.3* coding region from MGAS315 was cloned into the *E. coli*/GAS shuttle vector pJRS525 (McIver and Scott, 1997), generating plasmid pSL501. PCR mutagenesis was used to convert the internal TAA stop codon into a GAA glutamate codon, resulting in plasmid pSL502 with continuous full-length *scl1.3FL* allele. This sequence was subsequently cloned into the pASK-IBA2 expression vector for recombinant Scl1.3FL protein production; each clone was verified by sequencing. Protein expression constructs are listed in Table 1.

**Table 1:** **Constructs used in this study**.

**Plasmid**	**Description**	**Source**
pJRS525	*E. coli* / *S. pyogenes* shuttle vector	McIver and Scott, 1997
pSL230	pJRS525 with *scl1.41* allele	Caswell et al., 2007
pSL501	pJRS525 with *scl1.3WT* allele	Flores et al., 2015
pSL502	pSL501 with repaired allele *scl1.3FL*	Flores et al., 2015
pSL518	pSL502 with Kan^R^ in place of Spc^R^	This study
pASK-IBA2	*E. coli* expression vector	IBA, Göttingen
pSL154	pASK-IBA2 encoding rScl1.3V	This study
pSL503	pASK-IBA2 encoding rScl1.3FL	Flores et al., 2015
pSL514	pASK-IBA2 with rScl2.3	This study
pSB027	GFP-encoding plasmid	Cramer et al., 2003

Protein expression was induced by the addition of anhydrotetracycline at 0.2 μg mL^−1^ for 3 h. Cells were centrifuged and resuspended in high sucrose buffer (100 mM Tris-HCl, 1 mM EDTA, pH 8.0, 500 mM sucrose) or Cell Lytic B Buffer (Sigma), for separation of the periplasmic fraction and subsequent affinity purification. Proteins were dialyzed against 25 mM HEPES, pH 8.0, and analyzed by SDS-PAGE stained with RAPID*stain*™ (G-Biosciences). Protein sequence was confirmed using mass spectrometry (University of Nebraska Medical Center) and N-terminal Edman Degradation sequencing (Iowa State University).

#### Electron microscopy of rotary shadowed rScl proteins

Electron microscopy visualization of the rotary shadowed preparations of rScl1.3FL and rScl2.3 was used to assess the domain organization of rScl proteins, as employed previously (Xu et al., 2002). The rScl proteins were dialyzed against 0.1 M ammonium bicarbonate and mixed with glycerol to a final concentration of 70% (vol:vol). Samples were nebulized onto mica chips with an airbrush and rotary-shadowed with carbon/platinum using an electron beam gun. Photomicrograph images were acquired using a transmission electron microscope FEI G2 operated at 80 KV.

#### Mass spectrometry analysis

Samples from in-gel trypsin digested proteins were cleaned using a Millipore μC18 ZipTip, then resuspended in 0.1% formic acid. Samples were fractionated on a Eksigent cHiPLC column (75 μm × 15 cm ChromXP C18-CL 3 μm 120 Å), and resulting peptides were sequenced using 5600 TripleTOF (typical gradient 2–60% ACN in 60 min). Peptides identified were searched against the NCBI protein database with Protein Pilot software employing the following settings: search effort, thorough; taxonomy, none. Positive identification was considered as the identification of two or more unique peptides at high confidence ≥95%, FDR = 0.05, 0.01, or 0.001, which matched the same protein entry in the database searched.

#### Binding of rScl proteins to extracellular matrix proteins and synthetic peptide

##### ELISA binding assays

Binding of rScl proteins to extracellular matrix proteins, cellular fibronectin (cFn) and laminin (Lm), was tested by ELISA (Caswell et al., 2010). rScl proteins were immobilized onto *Strep*-Tactin® coated microplate wells (IBA GmbH) at 0.5 μM at room temperature for 1.5 h, then blocked with Tris-buffered saline (TBS) containing 1% bovine serum albumin (BSA) overnight at 4°C. The cellular fibronectin from human foreskin fibroblasts (Sigma) and murine laminin (Invitrogen) were added to the wells at 1 μg per well and incubated at room temperature for 1 h. Bound ECM proteins were detected with rabbit anti-laminin at 1:100 (Sigma) and anti- human fibronectin at 1:4000 (Sigma) polyclonal antibodies. Secondary antibody goat anti-rabbit IgG (H+L) HRP conjugate (Bio-Rad) was next used with 1-step ABTS substrate (2,2′-Azinobis [3-ethylbenzothiazoline-6-sulfonic acid]-diammonium salt) (ThermoScientific). Absorption was measured using Spectramax 190 at a wavelength of 415 nm. Statistical analysis is based on three independent experiments each containing three technical replicates, using an unpaired *t*-test.

In antibody inhibition assay, the IST-9 mAb targeting the C-C′ loop of EDA domain was utilized (Oliver-Kozup et al., 2013). cFn was either untreated or pre-treated with increasing concentrations of IST-9 blocking mAb (0.1–1.0 μg), and added to *Strep*-Tactin®-coated microplate wells immobilized with rScl proteins, then incubated for 1 h. Bound ECM proteins were detected as above.

##### Design and synthesis of the C-C′ cyclic peptide

The C-C′ cyclic peptide was designed based on the crystal structure of the EDA domain of cFn (PDB code 1J8K). In particular, the region, which was reported to be involved in Scl1 binding, connecting to two β-strands C and C′ of EDA was elongated to the whole C-C′ β-hairpin by allowing the formation the electrostatic interaction between Arg33 and Glu45. The terminal Tyr32 and Pro48 were mutated to Cys to stabilize the β-hairpin by introducing a disulfide bond (Figure S4). The obtained sequence, **C**RVTYSSPEDGIHELF**C** (molecular weight: 1997.1 Da), endowed with a cyclic structure to mimic the structure of this region in EDA, was acetylated and amidated at the N- and C-terminus, respectively. Synthesis of the designed peptide was performed employing the solid phase method on a 50 μmol scale initially following standard Fmoc strategies (Fields and Noble, 1990). Due to aspartimide formation during traditional acylation reactions, peptide synthesis was carried out employing microwave technology (Vanier, 2013). Cyclization was achieved by treating the peptide at 0.1 mg/mL (to avoid intermolecular disulphide formation) with buffer carbonate 50 mM, pH = 9, overnight. The peptide was purified by RP-HPLC and the identity and purity (>97%) was assessed by LC-MS (data not shown).

##### Surface plasmon resonance (SPR) experiments

Real time binding assays were performed at 25°C on a Biacore 3000 Surface Plasmon Resonance (SPR) instrument (GE Healthcare). For immobilization, rScl1.3FL protein containing the C-terminal *Strep*-tag II was injected at a concentration of 40 μM on streptavidin-coated sensor chip, SA Biacore, until the desired level of immobilization was achieved (averaged value of 100 RU). Binding assays were carried out by injecting the C-C′ cyclic peptide at concentrations ranging between 10 and 500 μM. Experiments were carried out in HBS buffer (10 mM HEPES, 150 mM NaCl, 3 mM EDTA, pH 7.4). The association phase (k_on_) was followed for 270 s, whereas the dissociation phase (k_off_) was followed for 300 s. The reference chip sensorgrams were subtracted from sample sensorgrams. Experiments were carried out in duplicates. Kinetic parameters were estimated assuming a 1:1 Langmuir binding model and using version 4.1 Evaluation Software (GE Healthcare).

##### Fluorescence binding analysis

rScl1.3FL protein, at a concentration of 30 μM, was incubated with increasing concentrations of C-C′ cyclic peptide (0–300 μM) at 25.0°C, using an excitation wavelength of 298.0 nm and a fluorescence emission wavelength ranging from 300 to 400 nm. The acquisition parameters were set as follows: excitation and emission slits at 5 nm; 120 nm/min scan rate; 1.00 nm data interval averaging time at 0.500 s, PMT voltage at “high.” Fluorescence values were recorded at 333 nm, and subtracted from the fluorescence intensity of the ligand-free protein, generating −Δfluorescence. The −Δfluorescence values were plotted against the peptide concentration (Williamson, 2013). Experiments were carried out in duplicates. A control assay was carried out employing the buffer as titrant to assess that the dilution effect was under 3%, not affecting the results.

### Complementation of GAS strains with full-length Scl1.3FL

For *trans*-complementation experiments, plasmid pSL502, encoding full-length cell-associated Scl1.3FL protein, was electroporated into MGAS315 WT and MGAS6183Δ*scl1* electrocompetent cells. The pJRS525 vector was electroporated as a control. Transformants were selected on BHI agar containing 100 μg mL^−1^ spectinomycin, and plasmids were re-sequenced. For MGAS10870Δ*scl1*, which contains a spectinomycin resistance cassette in place of the *scl1.3* allele, the spectinomycin resistance marker in pSL502 was replaced with a kanamycin resistance, generating the plasmid pSL518 and colonies were selected on BHI agar containing 150 μg mL^−1^ kanamycin.

#### Determination of Scl1.3- and Scl2.3-protein expression in wild-type and complemented GAS strains

##### Western blot analysis

Expression of the Scl1.3 and Scl2.3 proteins was determined by western immunoblotting of the bacterial cell wall and culture supernatant protein fractions, as described before (Lukomski et al., 2000, 2001). Briefly, bacterial cultures were grown to an OD_600_ of 0.5 and cells were harvested by centrifugation. Culture supernatant proteins were precipitated with trichloroacetic acid (Sigma) to a final concentration of 10% (vol:vol). The cell wall protein fraction was obtained after cell digestion with lysozyme and mutanolysin in a high sucrose buffer. A total of 10 μg of protein samples were separated by SDS-PAGE and transferred to a nitrocellulose membrane. Detection of Scl1.3, Scl2.3, and M3 proteins was performed using the same sample preparations with rabbit polyclonal antibodies generated against the truncated rScl1.3WT protein (anti-Scl1 1:15,000 dilution, reported in Flores et al., 2015) and the rScl2.3V region (anti-rScl2.3V 1:2500; generated by Proteintech Squeglia et al., 2014). Horseradish peroxidase-conjugated goat anti-rabbit IgG (H+L) secondary antibody (Bio-Rad), combined with Pierce^TM^ ECL western blotting substrate (Thermo Scientific) was used for detection. Images were acquired using a ChemiDoc Touch Imaging System (Bio-Rad).

##### Flow cytometry analysis

Surface detection of Scl1.3 and Scl2.3 proteins was determined by flow cytometry. Bacteria grown to an OD_600_ of 0.5 were harvested by centrifugation, and washed with flow cytometry buffer (sterile phosphate-buffered saline containing 10% Todd-Hewitt broth supplemented with 0.2% yeast extract). Cells were incubated with polyclonal antibodies against Scl1.3 and Scl2.3 described above at a dilution of 1:100 for 30 min on ice, then washed and incubated with Allophycocyanin (APC)-conjugated donkey anti-rabbit IgG (H+L) (Jackson ImmunoResearch) for 30 min on ice. Cells were washed and fixed in 0.4% paraformaldehyde, and stored at 4°C until analysis. Before analysis, cells were washed twice and resuspended in flow cytometry buffer. Cells were analyzed using a BD LSRFortessa, and 50,000 events were collected per sample. Data was analyzed using the FCS Express Flow 5 software.

### Assessment of biofilm formation

#### Crystal violet staining assay

Wild-type, mutant, and complemented strains were grown to OD_600_ of 0.5 and seeded into 24-well culture plates coated with ECM at 2 μg per well, then incubated at 37°C with 5% CO_2_ for 24 h. Wells were washed with PBS followed by the addition of 0.5 mL 1% crystal violet solution (Fisher Scientific) diluted in PBS and incubation at room temperature for 30 min. Wells were rinsed twice with PBS and stain was solubilized with 0.5 mL of 75% ethanol. Spectrophotometric readings were taken for each sample at OD_600_. Statistical analysis is shown based on three independent experiments, each containing three technical replicates, using an unpaired *t*-test.

#### Confocal laser scanning microscopy (CLSM)

To visualize GAS by CLSM, bacterial cells were transformed with a GFP-encoding plasmid pSB027 (Cramer et al., 2003), as before (Oliver-Kozup et al., 2011). Fifteen millimeter glass cover slips were placed into 24-well tissue culture plate wells and coated with 2 μg of cFn or Lm per well. Bacterial cultures grown to logarithmic-phase were added to the wells and allowed to form biofilms for 24 h. Wells were rinsed with PBS and bacterial cells were fixed with 3% paraformaldehyde at room temperature for 30 min. Wells were washed again and coverslips were mounted onto slides using ProLong Gold Antifade Mountant (Thermo Scientific). Confocal images were acquired using a 63 × /1.40 Plan-Apochromat objective and a Zeiss LSM 510 laser scanning confocal microscope.

### *In vitro* and *in vivo* GAS infection models

#### GAS infection of *in vitro* cultured human skin equivalents

Wounded full-thickness skin equivalents, EpiDerm-FT (MatTek, Boston, MA) were used. The tissues are discs 8 mm in diameter, which are provided in transwells. A 3-mm wound is generated by performing a punch biopsy to remove the keratinocyte layer. Immediately upon arrival, tissues were equilibrated in antibiotic-free manufacturer's medium overnight at 37°C in atmosphere with 5% CO_2_. Wounds were infected with 10 μL of GFP-expressing log-phase group A streptococcal inocula and incubated in a humid environment at 37°C with 5% CO_2_ in daily-fresh media; a total of 4 experiments were performed and variables included the inoculum size of 3 × 10^6^ − 1.8 × 10^7^ CFU and collection time points between 1 and 5 days. Tissues designated for histopathological evaluation were fixed in 10% formalin, whereas tissues for two-photon fluorescence (TPF) microscopy imaging were fixed in 4% paraformaldehyde for several hours and then transferred to petri dishes containing PBS. For visualization of glycocalyx produced by GAS strains, tissues were permeabilized with 0.1% Triton X-100 in 1 × PBS and blocked with 0.05% Triton X-100 in 1 × PBS with 1% BSA before staining with concanavalin A- tetramethylrhodamine (Molecular Probes). Tissues were then rinsed and stored in PBS at 4°C until TPF imaging was performed. For TPF analysis, an Olympus 60 × /1.2NA water dipping objective was used. Fixed tissues were imaged by two-photon microscopy with the Ti:sapphire laser (Mira, Coherent) intensity at 60 mW and input wavelength of 850 nm. Laser scanning images were collected at 0.5−1 μm incremental depths using ScanImage (Janelia Farms, HHMI). Images were saved in a single TIFF file with 16 bit depth. Deconvolution of the images was performed using AutoQuant × 3 and 3D models of z-stacks were generated using Imaris software.

#### Mouse model of soft tissue infection

Animal experiments were conducted in compliance with the regulations and standards under the Animal Welfare Act, the Public Health Service Policy on Humane Care and Use of Laboratory Animals, and the Guide for the Care and Use of Laboratory Animals. The protocol was approved by the West Virginia University Institutional Animal Care and Use Committee (IACUC).

Subcutaneous infections of mice were carried out as described previously (Lukomski et al., 1999). Briefly, 5-week-old male, immunocompetent, hairless mice (strain Crl:SKH1-*hr*BR) were used (Charles River, Wilmington, MA). Groups of 10–15 mice anesthetized with isoflurane were infected subcutaneously at the right flank with ~10^9^ GAS CFU of WT or *scl1*-mutant strains, and mice were observed for 14 days. The weight and abscess dimensions (length [L] and width [W]) of each mouse were recorded daily during the first week and every other day thereafter. To analyze differences between mice infected with WT and *scl1*-mutant GAS, the area of each abscess was calculated with the equation for the area (A) of an spherical ellipsoid: A = π(L/2) × (W/2); statistical differences were calculated using the student's *t*-test. At the conclusion of the experiments, mice were anesthetized and sacrificed by cervical dislocation.

## Results

### M3-type GAS contain unique insertion of IS1548 element and nonsense mutation within the *scl1.3* locus

Since the resurgence of invasive GAS infections in the 1980s and an advent of molecular epidemiology fostered by large-scale sequencing, significant efforts have been made to define the molecular basis for the invasive phenotype of M3-type strains. In parallel to these advances, we identified two unique genomic traits in the *scl1.3* locus, encoding streptococcal collagen-like protein 1: (i) the presence of an insertional sequence element, IS1548, in the promoter region and (ii) the presence of a nonsense mutation within the coding sequence of *scl1.3* allele that was absent in other M-types.

**Figure 1:**
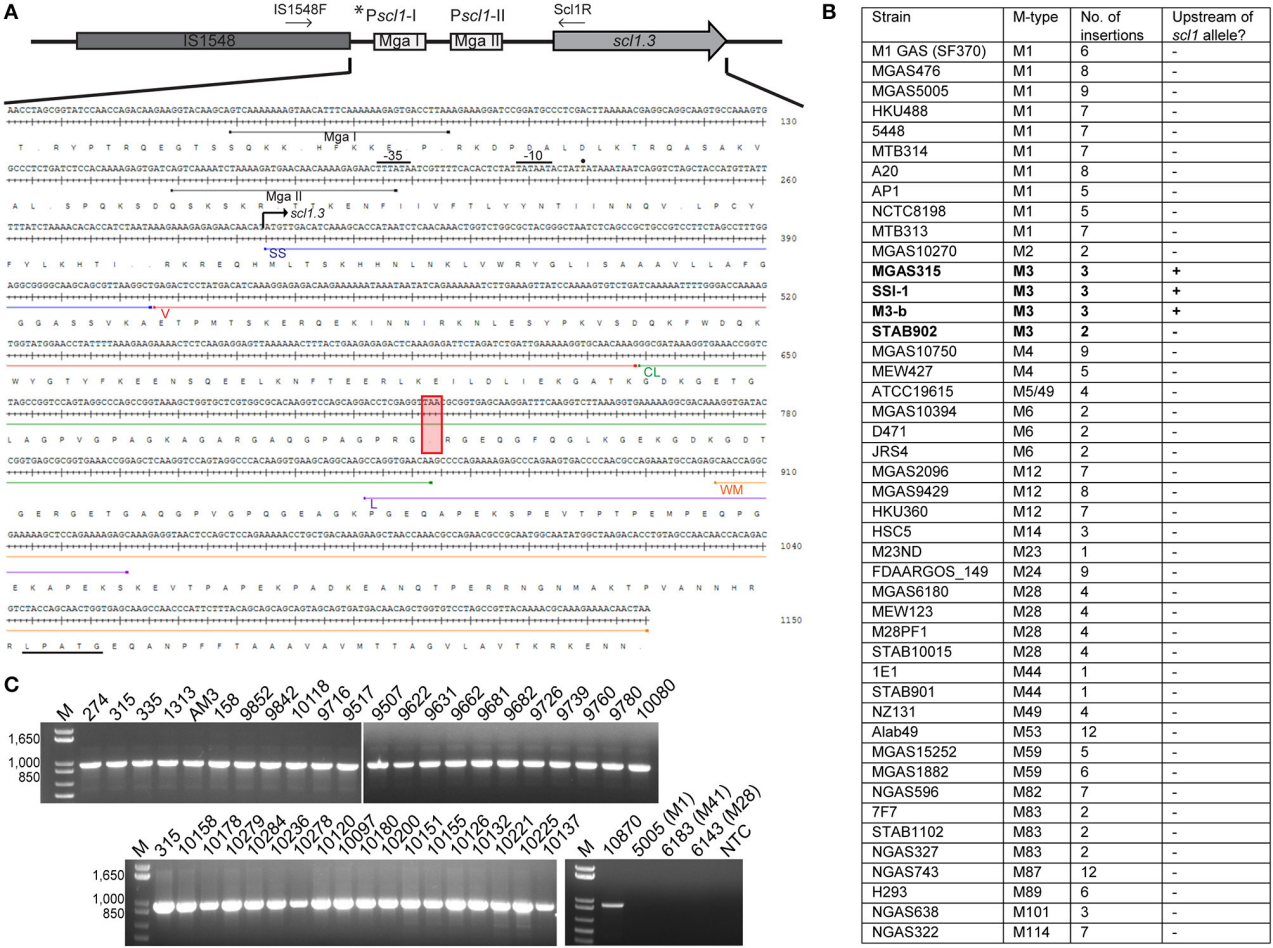
**M3-type GAS strains harbor unique polymorphisms in the *scl1.3* locus. (A)** Schematic representation and nucleotide sequence are based on the *scl1.3* locus in the M3-type strain MGAS315 genome. The *scl1.3* promoter region contains two putative Mga binding sites, P*scl1*-I with Mga I and P*scl1*-II with Mga II. IS1548 is inserted 38 bp upstream of the preferred ^*^P*scl1*-I/Mga I promoter, which was shown to be necessary for *scl1* transcription. Transcriptional start site (solid dot), −10 and −35 boxes are shown upstream of *scl1.3* coding sequence. Scl1 regions are designated as follows: SS, signal sequence; V, variable region; CL, collagen-like region; L, linker region; WM, wall-membrane region; LPATG, cell-wall anchor. The null mutation in the 11th Gly-X-Y repeat of the CL region is depicted by the red box, presumably resulting in a truncated secreted Scl1.3 protein. Relative location of primers, IS1548F and Scl1R, used to generate amplicons in **(C)** are shown. **(B)** IS1548 insertion upstream of *scl1.3* is unique to M3 genomes. BLAST search in the NCBI nucleotide (nr/nt) database using IS1548 (1317 bp) sequence as query identified insertions in 45 GAS genomes representing 21 different M-types. Only genomes of M3-type strains harbored IS1548 element upstream of the *scl1* allele (bold text). **(C)** IS1548 insertion upstream of *scl1.3* is conserved among M3 strains. Genomic DNA was isolated from a collection of 40 M3-type strains and analyzed by PCR for the presence of IS1548 upstream of *scl1* using primers IS1548F and Scl1R (located in conserved *scl1* signal sequence). Additional M1-, M41-, and M28-type control strains, and a no template control (NTC) are included. Expected amplicon size, 963 bp; M, 1 kb Plus DNA Ladder. MGAS designation applies to all strain numbers shown above gel wells, with the exception of strain AM3.

Studies employing *mga*-inactivated mutants have indicated that Scl1 expression was positively regulated by the GAS-global transcriptional regulator, Mga (Rasmussen et al., 2000; Lukomski et al., 2001). Two putative Mga binding sites were identified upstream of the *scl1* coding sequence in the M1-type strain SF370 and experiments demonstrated that the Mga binding site distal to *scl1* was responsible for transcription activation (Almengor and McIver, 2004). Identical Mga binding sites I and II were also identified upstream of *scl1.3* in the sequenced M3-type strain MGAS315 with IS1548 element inserted 38 bp upstream of the distal Mga I-binding site (Figure 1A). To determine if the IS1548 insertion was specific to M3 strains, we BLAST-searched this element in 45 completed GAS genomes representing 21 different M-types. We observed the presence of IS1548 in all strains searched with varying locations and occurrences from 1 to 12 per genome (Figure 1B). However, the IS1548 insertion upstream of the *scl1.3* allele was only found in the sequenced genomes of M3-type strains MGAS315, SSI-1, and M3-b. Interestingly, a complete IS1548 element was not present upstream of *scl1.3* in the recently reported genome of the M3 strain STAB902, which represents a non-invasive isolate (Soriano et al., 2014); instead, a 34-bp remnant of IS1548, including the inverted repeat and additional 14 bp, was found. Based on this bioinformatics data, we examined the presence of the IS1548 element upstream of *scl1* by PCR in a panel of 40 M3-type strains, using primers located in the IS1548 and *scl1.3* sequences (IS1548F and Scl1R, Table S1). All M3-type strains examined were positive for the IS1548-*scl1.3* amplicon, while M1, M41, and M28-type controls were negative (Figure 1C), demonstrating a broad and conserved presence of the IS1548 insertion in this location among M3-type GAS.

The nonsense mutation in the 11th Gly-X-Y repeat in the Scl1.3-CL collagenous region (Figure 1A, red box), presumably results in a truncated secreted variant, which consists of the Scl1-V variable region and 10 Gly-X-Y repeats but lacks the cell wall anchor. This polymorphism was originally identified in five M3-type invasive GAS strains but was absent in 45 other strains analyzed, representing 20 different M-types (Lukomski et al., 2000). It was later shown in 98.7% of 479 sequenced invasive M3-type isolates (Flores et al., 2015). We performed targeted PCR amplification of *scl1.3* from 46 additional M3-type strains and observed identical amplicon sizes in all of them (Figure S1A). Sequencing showed that all tested strains harbored an identical *scl1.3* allele, containing 25 Gly-X-Y repeats in the collagenous domain with the null mutation in the 11th repeat (Data Sheet 1). A complete lack of genetic variation within *scl1.3* is surprising and differs from the length variation that is commonly observed among *scl* alleles from other M-types (Lukomski et al., 2000; Rasmussen et al., 2000; Whatmore, 2001). These results demonstrate that the IS1548 insertion and null mutation we identified in the *scl1.3* locus are unique to and conserved among M3-type GAS. Based on these results, we hypothesized M3 strains produce a truncated, but potentially biologically active Scl1.3 variant, which is secreted instead of being cell-attached.

### *scl1.3* expression is diminished in M3-type GAS

**Figure 2:**
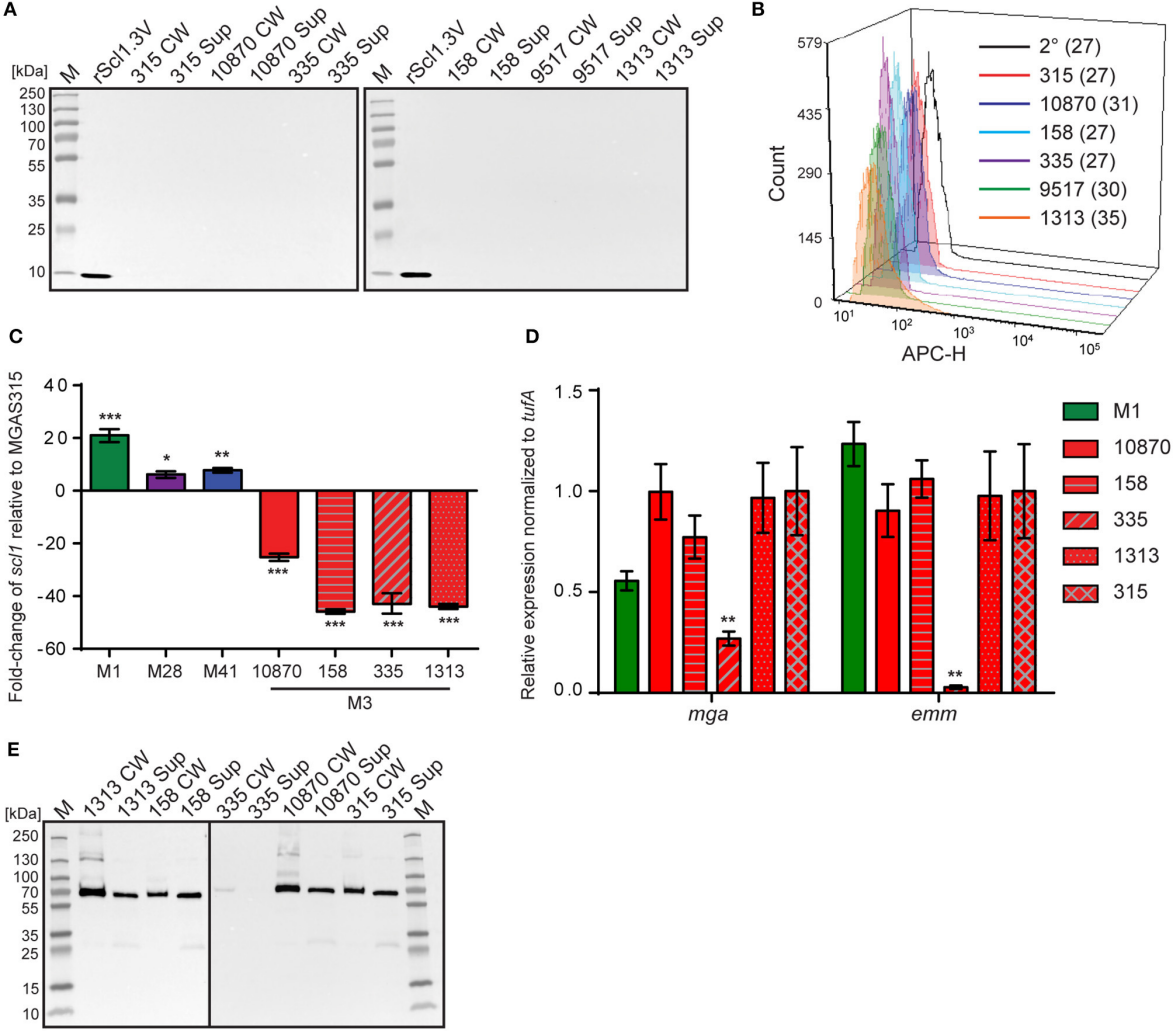
**Assessment of Scl1.3 expression. (A)** Assessment of Scl1.3 production by M3-type GAS. Cell wall (CW) and culture supernatant (Sup) protein fractions prepared from exponential phase cultures of several M3-type strains were analyzed by western immunoblotting, using anti-Scl1 rabbit polyclonal antibody. Recombinant protein rScl1.3V, corresponding to the variable region of Scl1.3, was used as a positive control. Expected molecular masses: Scl1.3, 11.4 kDa; rScl1.3V, 8.3 kDa. M, PageRuler™ Plus Prestained Protein Ladder. **(B)** Detection of Scl1.3 on the surface of M3-type GAS. Flow cytometry analysis of several M3-type strains is shown using anti-Scl1 antibody described in part **(A)** (color-shaded histograms) or a secondary only control (2° sample, black outlined histogram). Median fluorescence intensities (MFI) are shown in parentheses for each strain. **(C)** Assessment of *scl1* transcription. Fold-change of *scl1* transcript levels are shown compared to *scl1.3* transcription in M3-type strain MGAS315. qRT-PCR was performed on RNA obtained from exponential phase cultures. Results are shown from three independent experiments, each performed in triplicate wells. Standard errors and statistical analysis were computed from averaged ΔCt values for each biological replicate prior to normalization against the endogenous reference gene *tufA*; ^*^P ≤ 0.05, ^**^P ≤ 0.01, ^***^P ≤ 0.001 (student's *t*-test). **(D)** Assessment of *mga* and *emm* transcription. Relative expression levels of *mga* and *emm* genes were compared between MGAS315 and four additional M3 strains or the M1 strain MGAS5005. Results are shown from three independent experiments, each performed in triplicate wells. Standard errors and statistical analysis were computed from averaged ΔCt values for each biological replicate prior to normalization against the endogenous reference gene *tufA*; ^**^P ≤ 0.01. **(E)** Assessment of M3-protein production by M3-type GAS. The same cell wall (CW) and culture supernatant (Sup) protein samples prepared from exponential phase cultures of M3-type strains (used in **A**) were analyzed by western immunoblotting, using anti-M3 protein rabbit polyclonal antibody. Expected molecular mass: 65 kDa. M, PageRuler™ Plus Prestained Protein Ladder.

Original reports showed *scl1* transcripts in northern blots, as well as full-length Scl1 proteins (both cell-associated and cell-free fractions) in western blots for strains of *emm* types 1, 28, 52, and 41 (Lukomski et al., 2000, 2001; Rasmussen et al., 2000; Caswell et al., 2007). To analyze the expression of the truncated Scl1.3 protein in M3-type GAS, western blot analysis was performed on cell-wall (CW samples) and culture-supernatant (Sup samples) protein fractions of several M3-type strains grown to exponential phase (Figure 2A). The expected truncated Scl1.3 protein was not detected by anti-Scl1 antibodies, whereas the rScl1.3V positive control, corresponding to the V region of Scl1.3 variant, produced the expected immunoreactive band of ~8.3 kDa. In an additional control experiment, the same panel of M3 strains was tested for Scl1.3 on the cell surface using flow cytometry (Figure 2B). No shift in median fluorescence intensity was observed in M3 strains incubated with anti-Scl1 antibody compared to a secondary-only antibody control, indicating a lack of Scl1 on the cell surface among M3 strains.

Given the unique IS1548-*scl1.3* location and the lack of truncated Scl1.3 products in culture supernatants, we investigated *scl1.3* expression by qRT-PCR. Total RNA was isolated from exponential phase cultures of 5 M3-type strains, as well as from previously characterized control strains of *emm* types 1, 28, and 41 (Lukomski et al., 2000, 2001; Rasmussen et al., 2000; Caswell et al., 2007). Expression of *scl1* from each strain was compared to *scl1.3* transcription in M3 strain MGAS315 (Figure 2C). Each non-M3-type strain tested had significantly higher transcription level of respective *scl1* allele compared to MGAS315. The M1 strain had the most increased expression by 21-fold, while M28 and M41 strains exhibited 6- and 8-fold higher *scl1* expression, respectively. Interestingly, the *scl1.3* transcripts examined in four additional M3-type MGAS strains 158, 335, 1313, and 10870 were significantly reduced as compared to MGAS315, with a range of 25- to 45-fold decrease. Overall, we observed that M3-type GAS harboring the IS element upstream of *scl1.3* have drastically decreased *scl1.3* transcript levels and lack the Scl1 protein product, as assessed by western blot and flow cytometry.

Since *scl1* is regulated by the transcriptional activator Mga, we investigated whether the decreased *scl1.3* transcripts in M3 strains were due to lack of *mga* gene expression in these strains. In parallel, we assessed transcript levels of the *emm* gene encoding the M surface protein, a key virulence factor regulated by Mga. For comparison, we included the M1 strain, which had significantly increased *scl1* expression compared to the M3 strains. With the exception of MGAS335, which had significantly downregulated *mga* and *emm* expression, we found no significant differences in either *mga* or *emm* gene expression between MGAS315 and the other M3 strains or M1 strain (Figure 2D). Furthermore, M3 protein was highly expressed, as it was found in both cell wall and supernatant fractions, except for the MGAS335 strain, consistent with transcription data (Figure 2E). These results demonstrate that the striking downregulation of *scl1.3* in M3-type GAS is not due to decreased *mga* expression or non-functional Mga protein, as *emm* is normally expressed in these strains.

### *scl2.3* is expressed in M3-type GAS

Scl2 shares a similar structure with Scl1 but its biological function is poorly understood. One study demonstrated in a different M-type background that isogenic mutant devoid of Scl2.55 variant had lower adhesion to human skin fibroblasts (Rasmussen and Björck, 2001); however, the Scl2.3 variant present in M3-type GAS has not been investigated for expression and ECM binding. Therefore, we next assessed *scl2.3/*Scl2.3 expression in M3 strains. PCR amplification and sequencing showed that the majority of M3 strains contained in-frame *scl2.3* allele (Figure S1B, Table S2).

**Figure 3:**
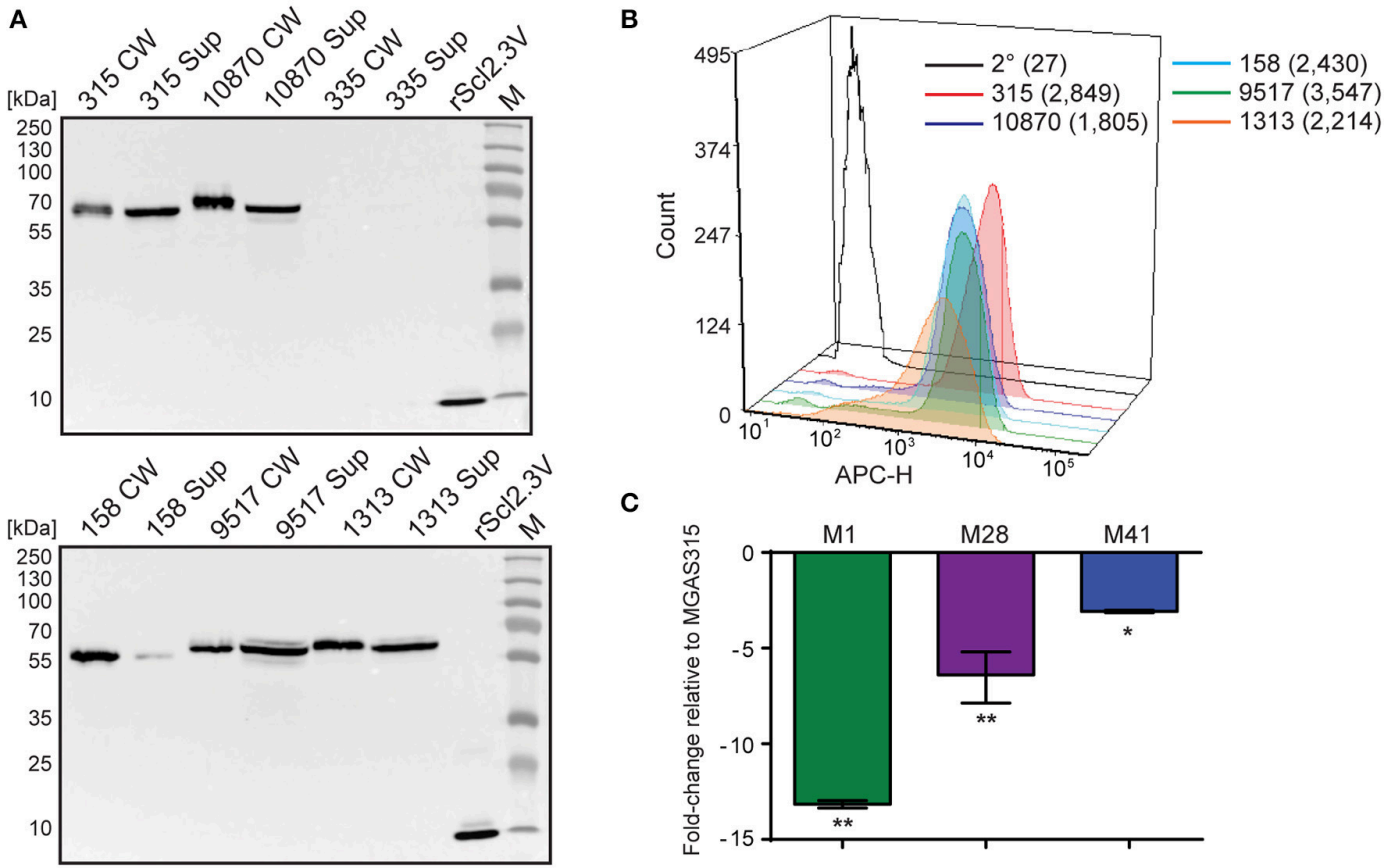
**Characterization of the *scl2.3* locus in M3-type GAS. (A)** Assessment of Scl2.3 production by M3-type GAS. The same cell wall (CW) and culture supernatant (Sup) protein samples prepared from exponential phase cultures of several M3-type strains (used in Figures 2A,E) were analyzed by western immunoblotting, using anti-rScl2.3V rabbit polyclonal antibody. Recombinant protein rScl2.3V, corresponding to the variable region of Scl2.3 protein, was used as a positive control. Expected molecular masses based on MGAS315: Scl2.3, 52.5 kDa; rScl2.3V, 10.1 kDa. Aberrant migration of detected Scl2.3 variants is characteristic of Scl proteins. M, PageRuler™ Plus Prestained Protein Ladder. **(B)** Detection of Scl2.3 on the surface of M3-type GAS. Flow cytometry analysis of several M3-type strains is shown using anti-rScl2.3V rabbit polyclonal antibody (color-shaded histograms) or a secondary-only control (2° sample, black outlined histogram). Median fluorescence intensities (MFI) are shown in parentheses for each strain. **(C)** Assessment of *scl2* transcription. Fold-change of *scl2* transcription levels are shown compared to *scl2.3* transcription in M3-type MGAS315. qRT-PCR was performed on reverse-transcribed RNA obtained from exponential phase cultures. Results are shown from three independent experiments, each performed in triplicate wells. Standard errors and statistical analysis were computed from averaged ΔCt values for each biological replicate prior to normalization against the endogenous reference gene *tufA*; ^*^P ≤ 0.05, ^**^P ≤ 0.01 (student's *t*-test).

Western blot analysis of cell wall (CW samples) and supernatant (Sup) protein fractions found Scl2.3 protein was expressed by M3 strains MGAS315, 10870, 158, 9517, and 1313, whereas samples obtained from strain MGAS335, which contains an out-of-frame *scl2.3* allele, generated no immunoreactive band; rScl2.3V control produced the expected 10.1-kDa band (Figure 3A). Mass spectrometry confirmed the identity of the presumed immunoreactive Scl2.3-protein band from MGAS315 (Table S3). Consistently, Scl2.3 was detected on the surface of all five M3 strains containing in-frame *scl2.3* alleles by flow cytometry, with a positive shift in median fluorescence intensity ranging in 67- to 131-fold change, as compared to the secondary-only antibody control (Figure 3B). We next compared the *scl2*-transcription level in M3 strain MGAS315 with *scl2*-transcription levels in M1-, M28-, and M41-type strains. In striking contrast to the pattern of *scl1.3* transcription, *scl2* transcripts were significantly decreased in the M1-type strain by 13-fold, as well as in the M28 (6-fold) and M41 (3-fold) strains (Figure 3C). Additionally, there was no significant difference between *scl2* expression in MGAS315 and the M3 strains MGAS158, 335, 1313, and 10870 (Table S4). These results confirm that the M3 *scl2.3* allele is transcribed at high levels, resulting in considerable expression of the Scl2.3 protein. These results suggested that Scl2 has an important biological function in M3 strains and warranted subsequent experiments assessing Scl2.3 function.

### GAS infection disseminates through human tissue and inhibits wound healing

**Figure 4:**
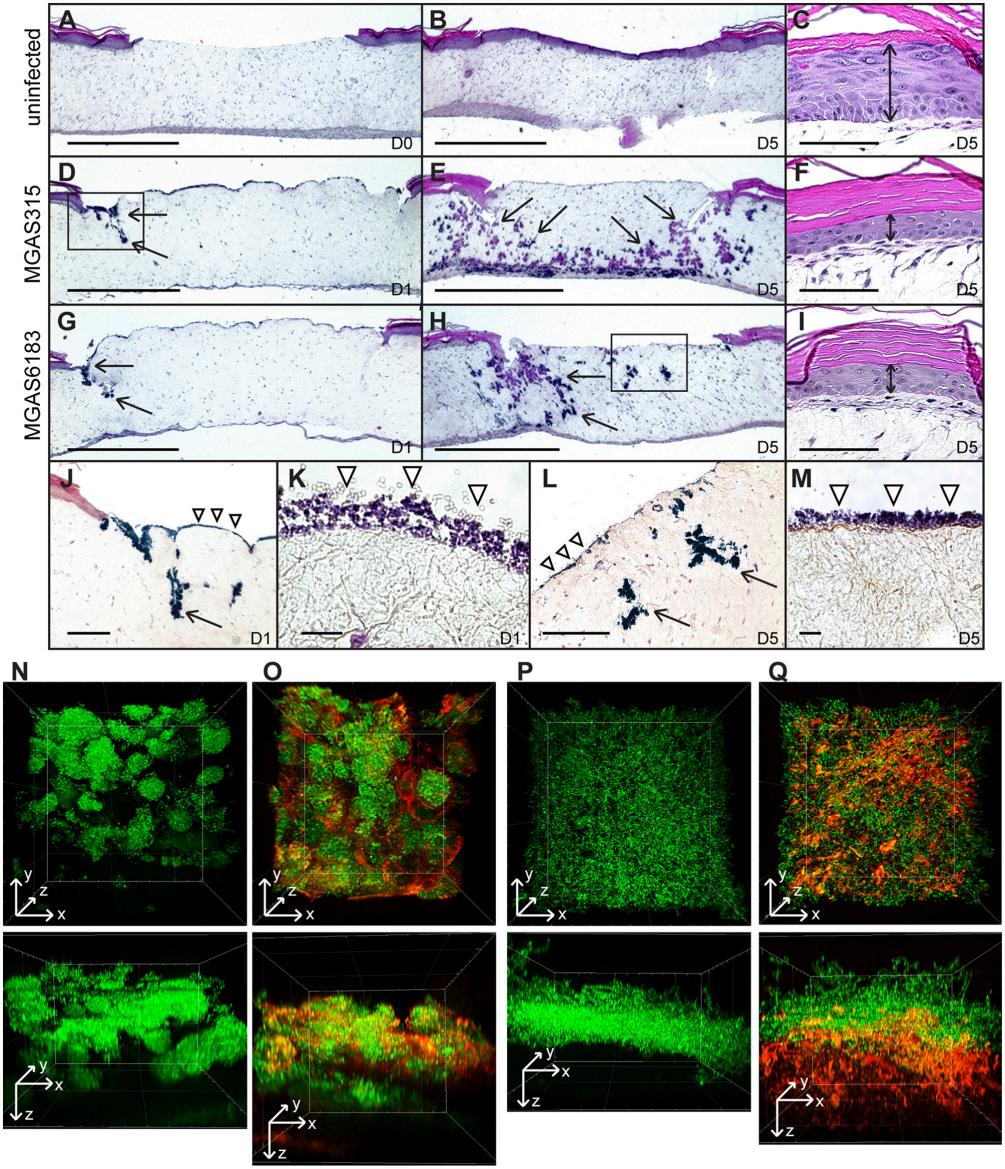
***In vitro* skin equivalent model of GAS infection. (A–I)** H&E stained sections of uninfected **(A–C)** or infected **(D–I)** wounded skin equivalents at 40x magnification; scale bar: 1000 μm. Uninfected wound at day 0 **(A)** shows a lack of the epidermal layer where biopsy punch was performed, which healed by day 5 **(B)**. At day 1, infection of wounded skin equivalents with M3 and M41 GAS revealed superficial colonization of the wound bed, as well as invasion into the defects formed at the wound edge (**D,G**, arrows). By day 5, bacteria had disseminated throughout tissue laterally and vertically (arrows), reaching the bottom of the dermis layer **(E,H)**. **(C,F,I)** H&E stained sections of the keratinocyte layer of intact skin surrounding the biopsy punch; scale bar: 100 μm. The thickness of the viable keratinocyte layer outside the wound (**C**, double arrow) was significantly reduced in tissues infected with M3 **(F)** and M41 **(I)** by day 5. **(J,L)** Gram stained sections of wounded tissue, corresponding to boxed areas in **(D,H)**, show tissue microcolonies (arrows) and superficial bacterial colonization (arrowheads). (**J**, scale bar: 400 μm; L, scale bar: 200 μm). **(K,M)** 1000x magnification micrographs of surface biofilms shown in **(J,L)** (scale bar: 10 μm). **(N–Q)** Two-photon fluorescence microscopy analysis of infected skin equivalents. Vertical dissemination through the wound bed and glycocalyx formation by the GFP-expressing M41- **(N,O)** and M3-type **(P,Q)** GAS at day 5. 3D projections of z-stacks are shown from the top view of the z-stack (top panels) or the side view (bottom panels). Multi-channel images show GFP fluorescence of GAS cells **(N,P)** and TRITC-conA stain of glycocalyx **(O,Q)**. All images were acquired at 600x magnification.

A wounded human skin equivalent, devoid of an inflammatory component, was utilized as a “mechanistic model” of GAS tissue colonization. The epidermal wound of each skin equivalent was infected with GFP-expressing M3-type invasive strain MGAS315 or M41-type non-invasive strain MGAS6183, and analyzed after 1–5 days by standard histopathology (H&E and Gram's stain) and using TPF microscopy. H&E of uninfected tissue controls harvested at day 0 showed the absence of a keratinocyte layer where the punch biopsy was performed (Figure 4A). Complete healing of the wound was observed after 5 days, with a newly-generated intact keratinocyte layer covering the punch biopsy site (Figure 4B). In contrast, tissue infected with either GAS strain exhibited delayed wound closure as late as day 5 post-infection (Figures 4E,H). H&E staining of skin equivalents infected with either M3 or M41 after 24 h revealed bacterial colonization of the exposed dermal surface, as well as bacterial invasion into puncture-associated defects extending deep into the dermal layer, largely located at the wound edges (Figures 4D,G). Notably, extensive bacterial growth and spread was largely confined to tissue crevices in the dermis, whereas the presence of large bacterial colonies directly below the wound bed was rarely seen on microscopic examination. By day 5 of infection, bacterial invasion via these dermal defects extended to the bottom of the dermis for both strains, presenting both vertical and lateral spread of bacteria (Figures 4E,H). Gram stain of infected tissues showed the formation of superficial colonies near dermal surfaces, as well as biofilm formation on the surface of exposed dermis (Figures 4J–M). Additionally, epidermal tissue neighboring the wound bed exhibited ~60% decreased thickness of the viable keratinocyte layer in tissues infected with MGAS315 and MGAS6183 by day 5, relative to uninfected tissues (Figures 4C,F,I). This suggests bacterial infection of the wound affects epidermal cells distant from the site of infection in this model.

TPF analysis was the performed on whole infected skin equivalents on day 5 post-infection in order to assess bacterial spread directly below the wound bed. This method allowed us to visualize bacterial structures within tissue that were not apparent in H&E or Gram-stained sections. Tissue microcolonies were observed in samples infected with M41 GAS (Figure 4N), whereas M3 cells had a scattered appearance (Figure 4P). TRITC-concanavalin A (TRITC-conA) was utilized to visualize glycocalyx associated with bacteria. TRITC-conA stain colocalized with bacterial microcolonies formed by the M41 GAS (Figure 4O, Figure S3), indicating microcolonies were encased in a glycocalyx, much like a classic biofilm. In contrast, TRITC-conA stain was associated with scattered M3-GAS chains located at the bottom cell layer in MGAS315-infected skin equivalents (Figure 4Q). These results indicate that M41 GAS, but not the M3 GAS, forms microcolonies in the tissue during a human skin infection that are encased in a glycocalyx, consistent with the observation that M41, but not M3, GAS forms robust biofilm *in vitro* on ECM coatings. In addition, M3 GAS disseminates in a form of scattered chains or single cells through the tissue.

### M3-type GAS strains form poor biofilms on extracellular matrix coatings

**Figure 5:**
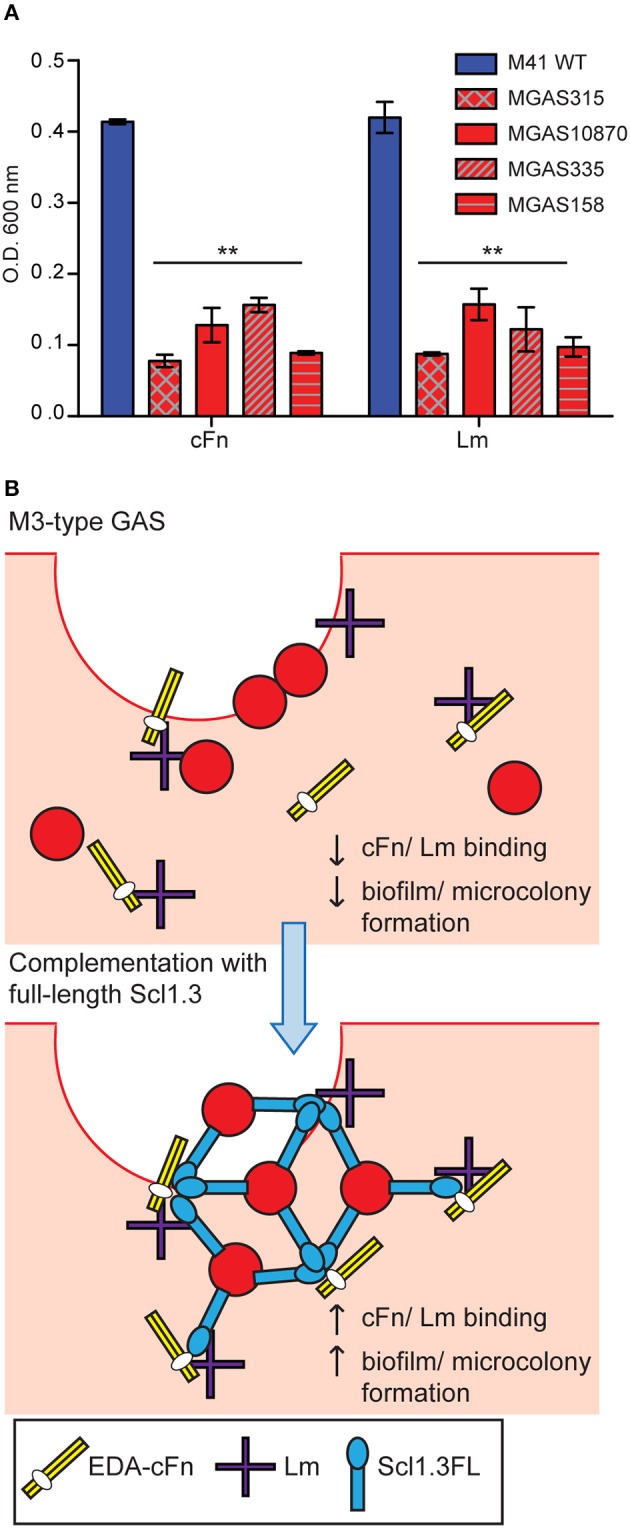
***In vitro* biofilm formation and hypothetical model of microcolony formation by invasive M3-type GAS. (A)** Limited *in vitro* biofilm formation by invasive M3 strains on coatings with cellular fibronectin and laminin. Invasive M3-type strains were compared to a non-invasive biofilm-capable M41-type strain MGAS 6183. Crystal violet staining was used to assess biomass formed after 24 h of growth in wells coated with cellular fibronectin (cFn) and laminin (Lm); results represent averaged values from at least 3 independent experiments performed in triplicate wells. ^**^P ≤ 0.01 (student's *t*-test). **(B)** Hypothesis model. Top: Infection of wounded skin with wild-type M3-type GAS. Inherent lack of surface-expressed Scl1.3 causes decreased binding to cFn and Lm expressed in wounded tissue, and reduces biofilm and tissue microcolony formation by M3-type bacteria (red circles). Bottom: *In-trans* complementation of M3-type GAS with full-length cell-associated Scl1.3, Scl1.3FL, restores binding to cFn and Lm in tissue, which confers biofilm and tissue microcolony formation during infection.

It was previously reported that M3-type strains have no substantial biofilm formation *in vitro* on an inanimate surface (Oliver-Kozup et al., 2011). Here, we tested biofilm formation on cellular fibronectin (cFn) and laminin (Lm) coatings by a panel of representative M3 strains isolated from invasive cases of GAS disease, as compared to the non-invasive biofilm-capable M41-type model strain MGAS6183. As expected, wells coated with either cFn or Lm supported robust biofilm formation by the M41 strain, whereas significantly less bacterial biomass was measured for all M3-type GAS on both ECM coatings (Figure 5A). There was also no correlation between Scl2 expression and biofilm formation. We hypothesized that M3-type GAS, devoid of Scl1 adhesin, have decreased binding to cFn and Lm ECM components, thus, preventing the formation of tissue microcolonies, and that restoration of full-length Scl1.3 (Scl1.3FL) on the GAS cell surface will confer binding to host ECM, as well as biofilm capacity *in vivo* (Figure 5B).

### Full-length recombinant Scl1.3 binds cellular fibronectin and laminin

To test this hypothesis, we: (i) constructed Scl1.3- and Scl2.3-derived recombinant proteins, (ii) characterized their structural organization, and (iii) assessed their ECM-binding capacities.

**Figure 6:**
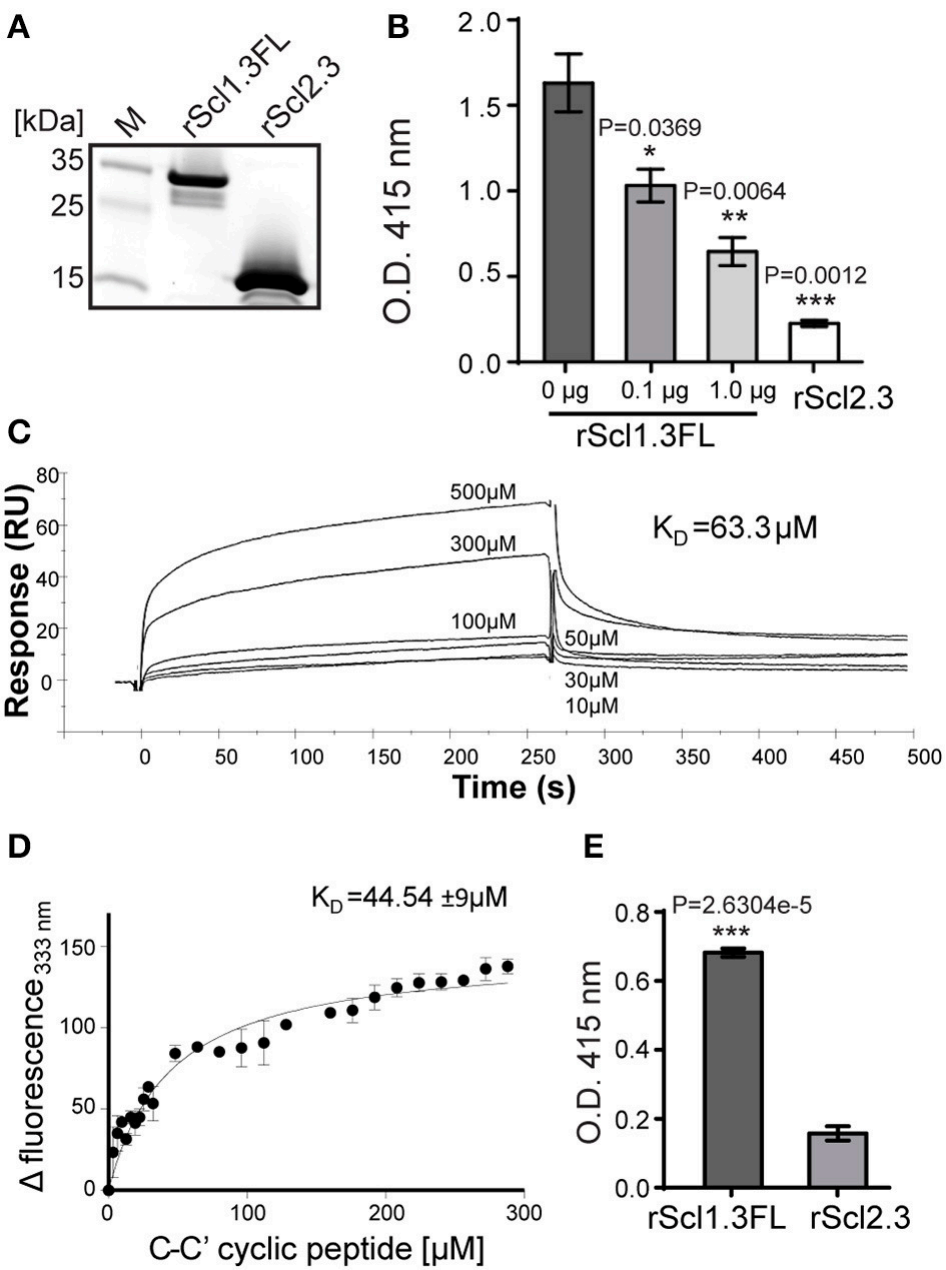
**Construction and binding characterization of recombinant full-length Scl1.3FL and Scl2.3 proteins. (A)** Affinity purified rScl1.3FL and rScl2.3 proteins were analyzed by 4–20% gradient SDS-PAGE; expected molecular masses: rScl1.3FL, 20.6 kDa and rScl2.3, 16.2 kDa. **(B–D)** Binding of rScl1.3FL to extra domain A (EDA) of cellular fibronectin. **(B)** IST-9 antibody inhibition identifies rScl1.3FL binding to the C-C′ loop of EDA-cFN. Inhibition of rScl1.3FL binding to EDA-cFn was tested by ELISA following pre-incubation of cFn with blocking IST-9 mAb specific to the C-C′ loop. Significance of inhibition by 0.1 and 1.0 μg of IST-9 mAb was determined by student's *t*-test as compared to the untreated cFn. A lack of significant cFn binding by rScl2.3 was evident, as compared to rScl1.3FL-cFn binding; student's *t*-test. **(C)** rScl1.3FL binding to a cyclic peptide mimicking the C-C′ loop of EDA using surface plasmon resonance. Overlay of sensorgrams for the interaction between immobilized rScl1.3FL and EDA-derived C-C′ cyclic peptide is shown. The experimental curves corresponding to different concentrations of peptide (10–500 μM) were fitted according to a single binding model with 1:1 stoichiometry. **(D)** rScl1.3FL binding to a C-C′ cyclic peptide using tryptophan fluorescence assay. Tryptophan fluorescence quenching analysis shows the dose-response curve of the fluorescence values of rScl1.3FL at 333 nm plotted against the concentration values of C-C′ cyclic peptide. **(E)** Laminin binding to rScl1.3FL and rScl2.3 by ELISA. Recombinant rScl proteins were immobilized onto Strep-Tactin-coated wells and incubated with laminin. Bound laminin was detected with specific primary pAbs and HRP-conjugated secondary Abs. Laminin binding was compared between rScl1.3FL and rSc2.3 and evaluated statistically using student's *t*-test. Results for **(B,E)** represent averaged values from at least 3 independent experiments performed in triplicate wells. ^*^P ≤ 0.05, ^**^P ≤ 0.01, ^***^P ≤ 0.001.

First, rScl1.3FL and rScl2.3 proteins were assessed for purity and integrity by SDS-PAGE (Figure 6A). The expected 20.6-kDa rScl1.3FL migrated at ~34 kDa, which is consistent with previous reports of aberrant migration of rScl proteins (Lukomski et al., 2000, 2001), whereas the rScl2.3 protein migrated according to the expected molecular mass of 16.2 kDa; both proteins were verified by mass spectrometry (Table S3). Rotary shadowed rScl1.3FL and rScl2.3 constructs, exhibited the characteristic lollipop-like structural organization (Figure S2), as observed for previously characterized rScl proteins (Xu et al., 2002; Han et al., 2006b). Interestingly, rScl1.3FL formed aggregates that were mediated by the intermolecular interactions between the globular domains; such interactions, however, were not observed between rScl2.3 molecules. The appearance of Scl1-Scl1 aggregates implies an attractive hypothesis that V-to-V-region interactions between the Scl1 molecules, but not between the Scl2 molecules, on the surface of neighboring GAS chains may support biofilm structure, as proposed in our model (Figure 5B).

It has been established that Scl1 proteins selectively bind cellular, but not plasma, fibronectin and laminin (Caswell et al., 2010), and that selective cFn binding is achieved by a unique binding mechanism involving the C-C′ loop of the extra domain A in cFn (EDA-cFn) (Oliver-Kozup et al., 2013). Here, we pre-incubated cFn with increasing concentrations of EDA-blocking IST-9 mAb (0.1, 1.0 μg), then added to wells containing immobilized rScl proteins and allowed for binding. We observed significant dose-dependent inhibition of cFn binding to rScl1.3FL, with a 36% inhibition at 0.1 μg and a 60% inhibition at 1.0 μg of IST-9 (Figure 6B). In addition, the rScl2.3 protein did not bind cFn, which is consistent with our previous data employing several different rScl2 proteins (Caswell et al., 2010). Surface plasmon resonance (SPR) measurements of binding affinity between rScl1.3FL and EDA-derived C-C′ cyclic peptide provided a dissociation constant of *K*_D_ = 63.3 μM (Figure 6C). To corroborate the rScl1.3FL-EDA binding affinity, an in-solution fluorescence binding assay was performed, in which the variation in tryptophan fluorescence of rScl1.3FL was recorded as a measure of positive binding to the C-C′ cyclic peptide. Tryptophan fluorescence emission at 333 nm showed a dose-response quenching upon addition of the C-C′ cyclic peptide and −Δ fluorescence intensity was plotted against peptide concentration (Figure 6D). Data were fitted with a 1:1 model of interaction, providing a *K*_D_ = 44.54 ± 9 μM, in agreement with SPR data (Russo et al., 2015).

We next assessed binding of rScl1.3FL and rScl2.3 proteins to laminin (Lm) by ELISA. We determined rScl1.3FL had significant Lm binding, whereas rScl2.3 had not (Figure 6E), consistent with previous findings that Scl1-derived recombinant proteins, but not the Scl2-derived, bind ECM proteins (Caswell et al., 2010). Collectively, these results demonstrate specific binding of Scl1.3FL to the EDA domain of cFn and to Lm and its capacity of being surface adhesin.

### Homologous complementation of M3 strains with full-length surface-exposed Scl1.3 adhesin confers biofilm formation on ECM

**Figure 7:**
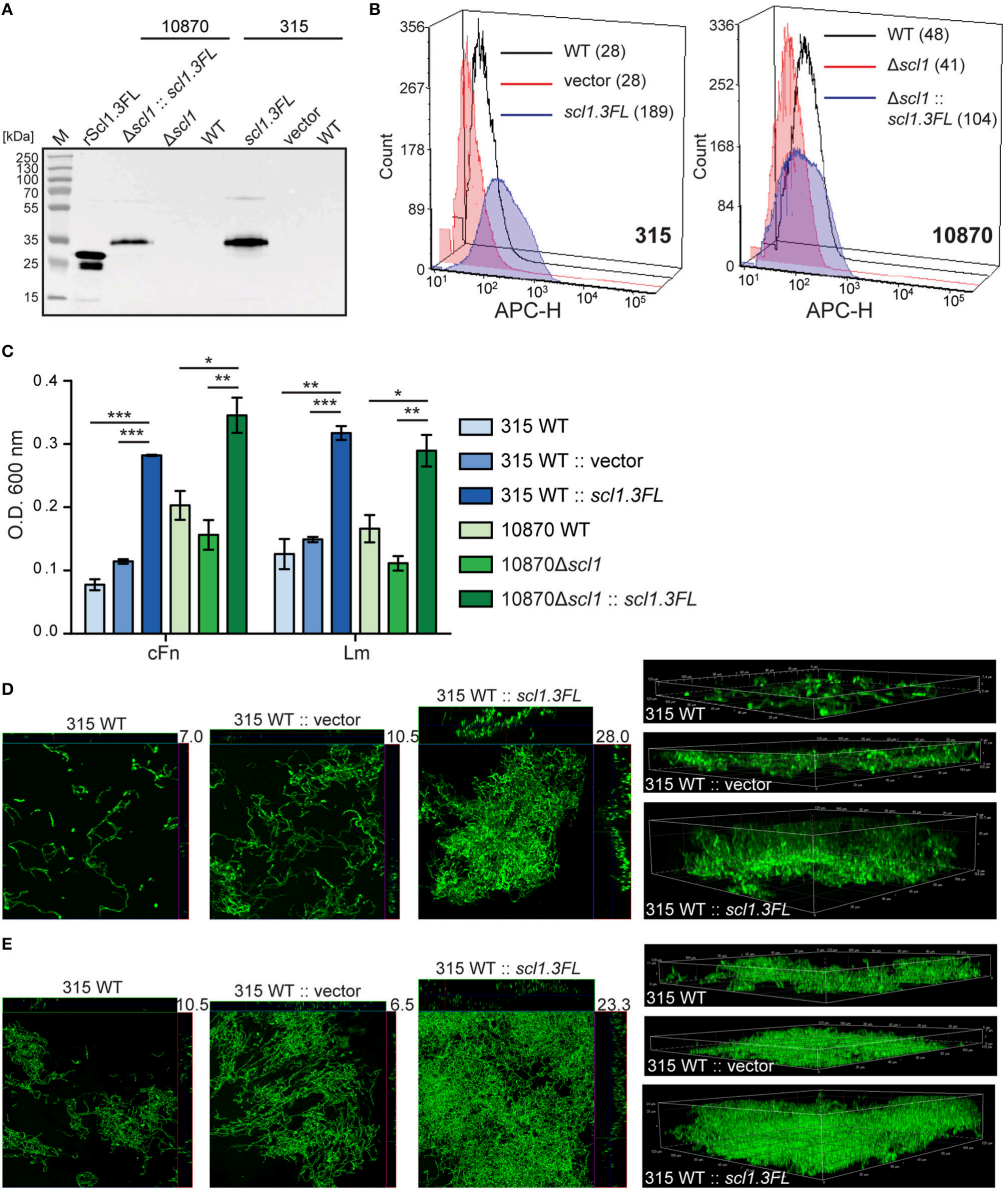
**Homologous complementation of M3-type GAS with full-length surface-expressed Scl1.3 protein confers biofilm formation. (A)** Western blot detection of full-length Scl1.3 in cell wall fractions of MGAS315 and MGAS10870Δ*scl1* complemented *in-trans* with the *scl1.3FL* allele. Parent strains were included as negative controls and rScl1.3FL was used as a positive control for detection. **(B)** Flow cytometry detection of Scl1.3FL on the GAS cell surface. Left: Fluorescence intensity of Scl1.3FL-complemented MGAS315 WT strain was compared to vector-complemented or WT parent strain. Right: Fluorescence intensity of Scl1.3FL-complemented MGAS10870Δ*scl1* was compared to WT and Δ*scl1* parent strains. Median fluorescence intensities are shown in parentheses for each strain. **(C)** Crystal violet assessment of bacterial biomass after 24 h of growth in cFn- or Lm-coated wells. MGAS315 WT complemented *in-trans* with pJRS525 (Spc^R^ vector) or pSL502 (Table 1) harboring the *scl1.3FL* allele was compared to WT parental strain. Spectinomycin resistant MGAS10870Δ*scl1* mutant was complemented *in-trans* with Kan^R^ pSL518 (Table 1), harboring the *scl1.3FL* allele. Biofilm biomass of the complemented strain was compared to MGAS10870 WT and *scl1*-inactivated parental strains. Results represent averaged values from at least 3 independent experiments performed in triplicate wells. ^*^P ≤ 0.05, ^**^P ≤ 0.01, ^***^P ≤ 0.001; students *t*-test. **(D,E)** Confocal laser scanning microscopy analysis of biofilm formation by GFP-expressing MGAS315 vector and *scl1.3FL*- complemented strains. Biofilms were grown for 24 h on cFn-coated **(D)** or Lm-coated **(E)** coverslips. Maximum intensity projections of GAS biofilms with cross-sectional views (left panels) are representative of z-stacks from 10 fields within a single experiment. Average vertical thickness is indicated in micrometers. 3D projections of z-stacks (right panels) are shown from the side view.

To assess the effect of cell-surface Scl1.3FL expression on the capacity to form biofilm, an *in-trans* complementation of two representative invasive M3 strains was performed, MGAS315 wild-type (WT) strain, naturally lacking Scl1.3 expression, and a previously generated *scl1*-inactivated mutant of MGAS10870 (10870Δ*scl1*), with plasmids pSL502 (Sp^R^) and pSL518 (Km^R^), respectively, both encoding the full-length Scl1.3FL protein. As a control, MGAS315 was complemented with a shuttle vector pJRS525. The cell wall-associated expression of Scl1.3FL in complemented M3-type GAS was first tested by western blot analysis of the cell wall protein fractions and on the GAS-cell surface by flow cytometry. An expected ~35-kDa immunoreactive band in complemented strains was observed, which was absent in the parent strains (Figure 7A). Mass spectrometry analysis of the corresponding bands extracted from the gel confirmed they represented the Scl1.3FL protein, with five unique peptides identified with 17% sequence coverage for complemented MGAS315 and four unique peptides identified with 11% sequence coverage for complemented MGAS10870 (Table S3). A 6.8-fold increase in median fluorescence intensity of Scl1.3FL-complemented MGAS315 cells was measured by flow cytometry, as compared to the vector-complemented and WT control strains (Figure 7B). The Scl1.3FL-complemented 10870Δ*scl1* exhibited a 2.2-fold increase in median fluorescence intensity, as compared to the mutant control (Figure 7B). These results indicate the Scl1.3FL is indeed expressed and surface-exposed in the complemented M3-type strains.

Biofilm formation by the complemented and parent strains was then assessed after 24 h following crystal violet staining and using confocal laser scanning microscopy (CLSM). Scl1.3FL-expressing MGAS315 showed significantly increased biomass on both cFn and Lm coatings compared to the WT parent organism, as well as vector-complemented control; 3.6 or 2.5- and 2.5 or 2.1-fold OD_600_ increases on cFn and Lm, respectively, were measured compared to MGAS315 WT or to vector-complemented MGAS315 (Figure 7C). Significantly thicker biofilm formed by Scl1.3FL-complemented MGAS315 was imaged by CLSM. We observed on average a 2.8-fold (*P* = 0.0002) and 2.6-fold (*P* = 0.0002) increased biofilm thickness on cFn and Lm, respectively, compared to MGAS315 WT, and a 2.0-fold (*P* = 0.0036) and 4.8-fold (*P* = 2.1 × 10^−5^) increased biofilm thickness on cFn and Lm, respectively, compared to vector-complemented MGAS315 (Figures 7D,E, representative images). Similar results were obtained for the complemented 10870Δ*scl1* mutant, which had 1.7-fold increase in biomass staining on both cFn and Lm compared to the MGAS10870 WT strain, and 2.2- and 2.6-fold increase in biomass staining on cFn and Lm, respectively, compared to the 10870Δ*scl1* mutant strain (Figure 7C). CLSM data, however, could not be rendered for MGAS10870 strains due to poor GFP expression for unknown reasons. Altogether, it was demonstrated that null mutation in the *scl1* gene, which ablates surface Scl1.3 protein and is unique to M3-type GAS, is responsible for the decreased biofilm capacity since restoration of the full-length surface-exposed Scl1.3 adhesin significantly fosters stable biofilm formation.

### Heterologous complementation of M41 Δ*scl1* mutant strain with full-length surface-exposed Scl1.3 restores biofilm formation on ECM

**Figure 8:**
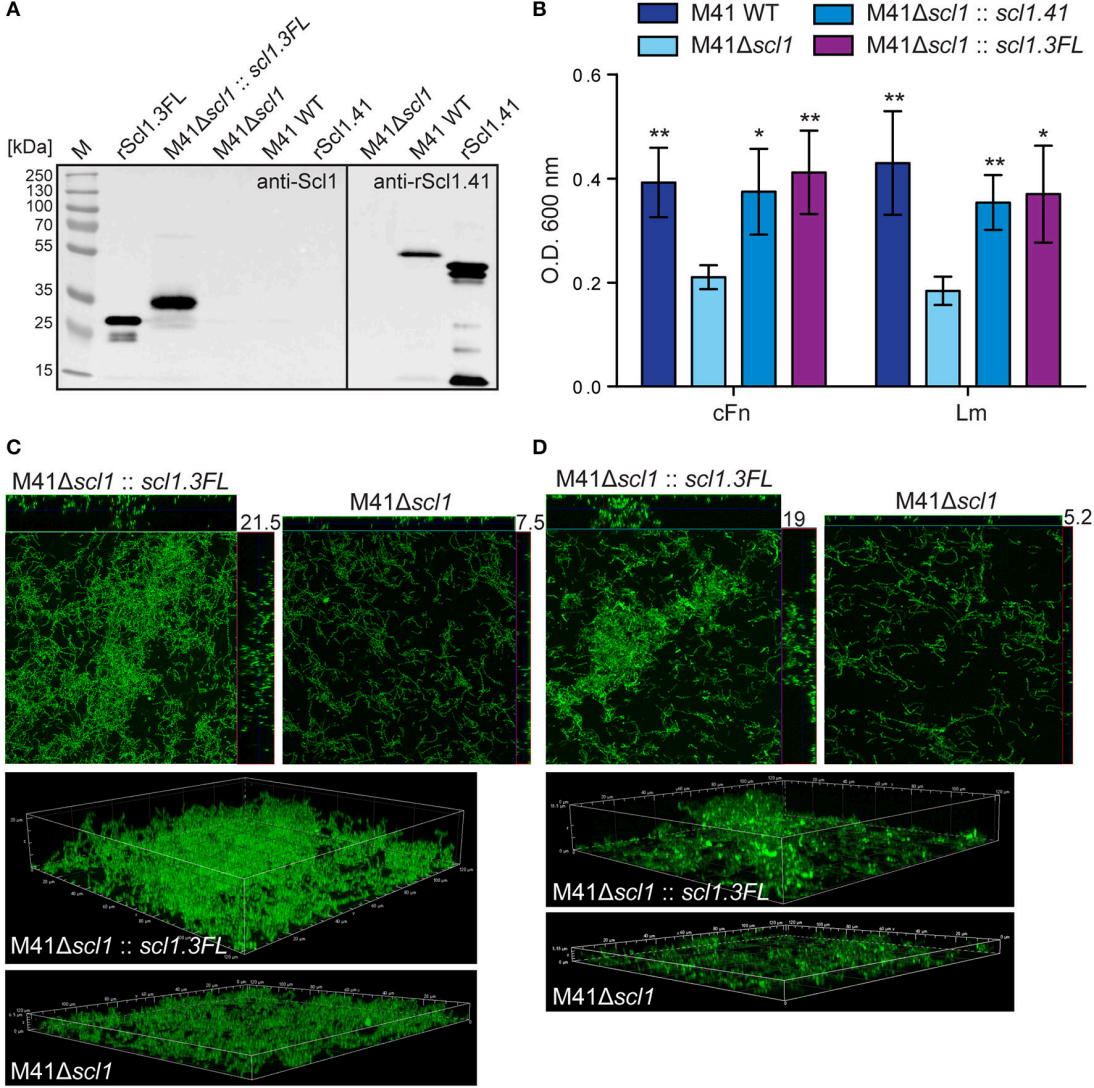
**Heterologous complementation of *scl1*-deficient mutant of M41-type GAS with full-length Scl1.3 restores biofilm formation. (A)** Left blot: western blot detection of full-length Scl1.3 protein in cell wall fraction of M41Δ*scl1*::*scl1.3FL* using anti-Scl1 polyclonal antibody. The M41Δ*scl1* and M41 WT strains were included as negative controls, and rScl1.3FL was included as a positive control. Right blot: western blot detection of Scl1.41 in M41Δ*scl1* and M41 WT strains using anti-rScl1.41 antibody and rScl1.41 as a positive control. **(B)** Crystal violet assessment of biofilm biomass after 24 h of growth in cFn- or Lm-coated wells. M41 *scl1*-inactivated (M41Δ*scl1*) mutant was complemented *in-trans* with either homologous *scl1.41* allele harbored on pSL230 or with heterologous *scl1.3FL* allele harbored on pSL502 (Table 1). M41 WT, Δ*scl1* mutant and two complemented mutant strains were assessed for biofilm formation and statistical significance was assessed by students *t*-test as compared to M41Δ*scl1*; ^*^P ≤ 0.05, ^**^P ≤ 0.01. **(C,D)** Confocal laser scanning microscopy analysis of biofilm formation by GFP-expressing M41Δ*scl1* or M41Δ*scl1*::*scl1.3FL*. Biofilms were grown for 24 h on cFn-coated **(C)** or Lm-coated **(D)** coverslips. Maximum intensity projections of GAS biofilms with cross-sectional views (top panels) are representative of z-stacks from 10 fields within a single experiment. Average vertical thickness is indicated in micrometers. 3D projections of z-stacks (bottom panels) are shown from the side view.

In a previous study we showed that *scl1.41*-inactivation in a non-invasive biofilm-rich M41 strain MGAS6183 resulted in significantly decreased biofilm capacity, which was restored to wild-type level by complementation with surface Scl1.41 (Oliver-Kozup et al., 2013). Notably, this M41 strain expresses at least one additional major Fn-binding protein, protein F2 (Caswell et al., 2007), which binds both plasma and cellular fibronectin by a mechanism different from Scl1 (Sela et al., 1993). Here, we hypothesized that expression of rScl1.3FL in the heterologous M41 GAS will confer biofilm formation. Western immunoblotting of the cell wall protein fractions detected the ~35-kDa immunoreactive band, corresponding to full-length Scl1.3, associated with complemented cells, while the M41Δ*scl1* mutant and WT cells were signal-negative (Figure 8A). Mass spectrometry of the corresponding band extracted from the gel confirmed Scl1.3FL expression, with 3 unique peptides identified, covering 13% of the amino acid sequence (Table S3). In addition, we showed the expression of the larger Scl1.41 variant in the cell wall of M41 WT, but not in the Δ*scl1* mutant, by re-probing a portion of the blot with anti-rScl1.41 antibody, using rScl1.41 protein as a positive control.

Similarly to complementation with homologous Scl1.41 protein, the heterologous complementation of the M41Δ*scl1* mutant with Scl1.3FL also restored biofilm to M41 WT levels on cFn and Lm (Figures 8B–D). Crystal violet staining showed increased bacterial biomass (Figure 8B) and confocal microscopy revealed significantly thicker biofilms, on average 3-fold increased, as compared to the parental M41Δ*scl1* mutant strain on both cFn (Figure 8C) and Lm (Figure 8D) coatings (cFn, *P* = 0.0105; Lm, *P* = 0.0011).

### Expression of Scl1 adhesin attenuates GAS during subcutaneous infection

We have previously shown that an M3 strain harboring a carrier *scl1.3* allele, producing a shorter cell-attached Scl1.3 variant, had an attenuated phenotype in a murine model of necrotizing fasciitis (Flores et al., 2015). Here, we tested our hypothesis that Scl1 adhesin in biofilm-rich M28 and M41 background promotes stable colonization and localized infection, using a murine skin infection model. Hairless, immunocompetent SKH1 mice were subcutaneously infected with ~10^9^ GAS CFU of the M28 and M41 wild-type (WT) or their isogenic *scl1*-inactivated mutant (*scl1*) strains (Han et al., 2006a; Caswell et al., 2007), and mice were assessed for changes in gross pathology of the skin.

**Figure 9:**
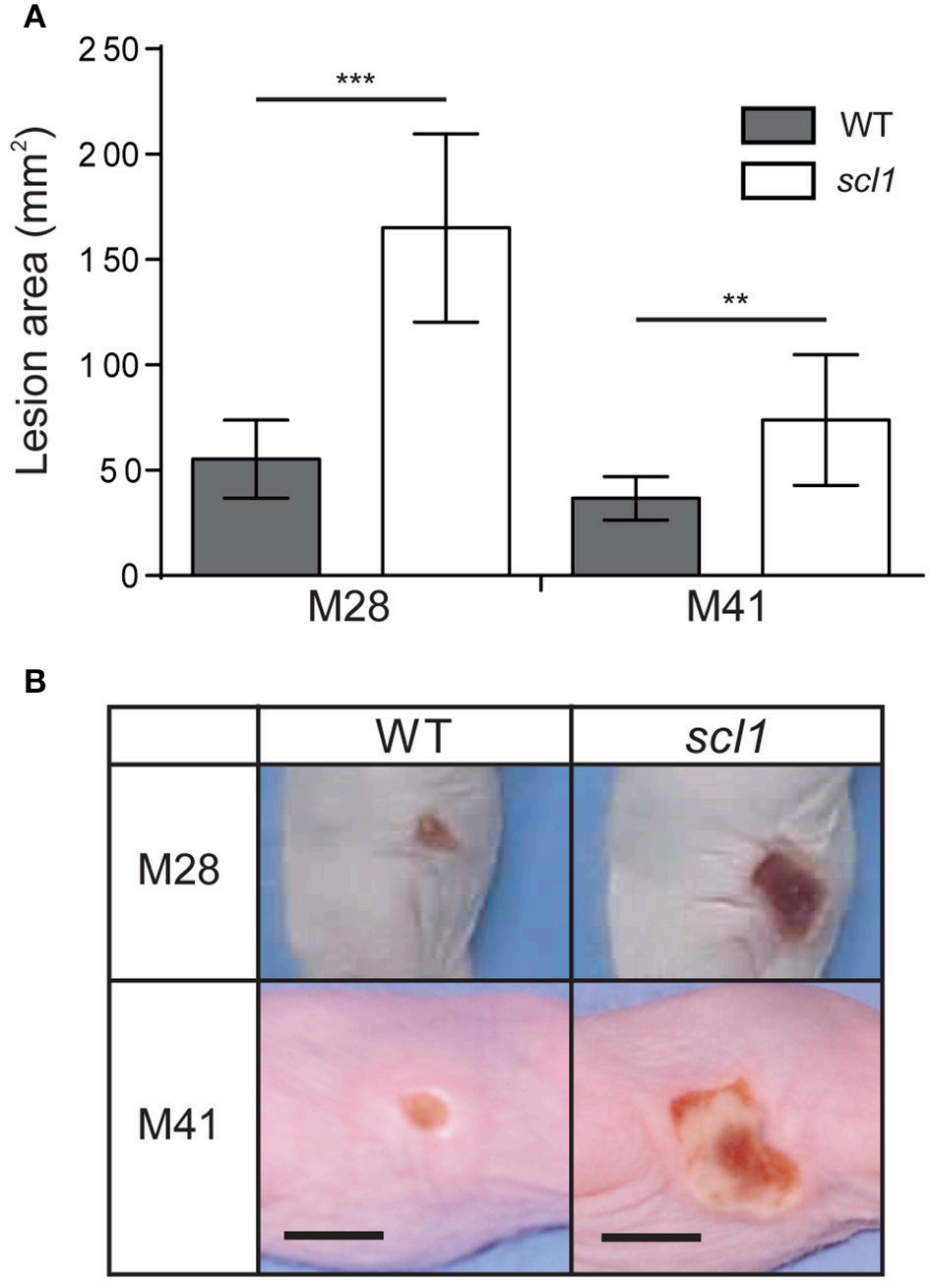
**Skin pathology of SKH1 hairless mice infected subcutaneously with wild-type and *scl1*-inactivated mutants of M28- and M41-type GAS. (A)** Mean area of skin lesions in mice infected with M28- or M41-type isogenic GAS strains. The severity of skin lesions was compared following infection with wild-type (WT) or *scl1*-mutant (*scl1*) GAS; 96-h time-point is shown. The mean lesion area and standard deviation were calculated and compared between experimental groups. Data represents mean lesion size from at least 10 mice per experimental group. Statistical differences were calculated using the student's *t*-test; ^**^P ≤ 0.01, ^***^P ≤ 0.001. **(B)** Gross pathology of the skin lesions. Digital images of the skin lesions of representative animals infected with either WT or *scl1*-mutant GAS. The images show skin lesions developed after 96 h post-infection, and scale bar represents 10 mm.

Skin lesions caused by both the WT and *scl1* strains were observed as early as 48 h post-infection, and the lesions began to regress after day 7 with complete resolution of the lesion by the completion of the experiment. The areas of the skin lesions calculated for mice infected with the *scl1* mutant strains were significantly larger than those of WT-infected mice (Figure 9A; 96 h time-point is shown); images of lesions of representative mice demonstrate differences in lesion severity between WT- and *scl1*-GAS infected mice for both the M28- and M41-infected groups (Figure 9B). Thus, the *in vivo* data, using GAS strains expressing surface Scl1 proteins and producing rich biofilms, support our hypothesis that decreased adhesion and biofilm formation, due to the absence of Scl1.3 on the surface of M3 strains, bears an inverse correlation to the invasive potential of the infecting GAS strain.

## Discussion

**Figure 10:**
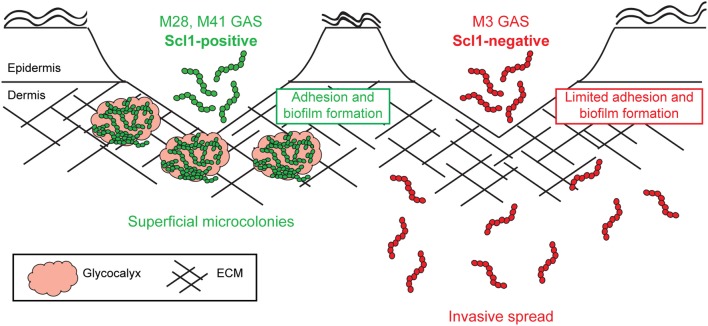
**Model of Scl1-mediated GAS adhesion, biofilm formation, and host colonization**. M28- and M41-type GAS express full-length cell-attached Scl1, which mediates adherence to cFn and Lm. ECM binding promotes biofilm formation in wounded tissue and allows the formation of superficial tissue microcolonies resulting in non-invasive colonization. On the contrary, in M3-type GAS, naturally lacking full-length cell-attached *Scl1.3* adhesin, bacterial cells have reduced adherence to cFn and Lm and reduced capacity for biofilm formation, thereby circumventing the formation of localized tissue microcolonies; infection balance is shifted toward dissemination through tissue, promoting invasive spread.

Since the resurgence of invasive GAS disease in the 1980's, and emergence of invasive M3-type isolates, significant efforts have been made to determine the molecular basis for the invasive phenotype of M3-type strains. Numerous whole-genome sequencing projects have identified specific genomic features of M3 strains that were correlated with their potential to cause invasive infections. In this study, we show that Scl1-negative M3-type GAS have reduced adhesion and biofilm formation within host tissue, and therefore are predisposed to invasive spread over superficial infection (Figure 10).

### Unique topography and expression patterns of *scl* loci

First, the insertion of IS1548 element in the promoter region of *scl1* exclusively found in the M3-type strains is an intriguing observation, given the abundance of IS1548 insertions across GAS genomes representing a variety of M-types. IS elements are known to cause genomic rearrangements and affect the expression of important genes that contribute to bacterial virulence (Mahillon et al., 1999). For example, the insertion of IS1548 element (Granlund et al., 1998) into the *scpB-lmb* intergenic region in group B *Streptococcus* has been shown to upregulate *lmb*-gene transcription and Lmb-adhesin surface expression, resulting in enhanced laminin binding (Al Safadi et al., 2010). The IS1548 insertion in the *scl1.3* promoter was conserved in the genomes of sequenced M3 strains MGAS315, SSI-1, and M3-b, as well as in 40 additional M3 strains tested by PCR. However, the recently sequenced STAB902 M3 strain (Soriano et al., 2014), which represents a non-invasive isolate, contained a 34-bp IS1548 remnant, suggesting that M3 circulating strains exist with this polymorphism. We hypothesize that the proximity of the IS1548 insertion to the Mga I binding site in M3 strains affects *scl1.3* transcription. Comparative qRT-PCR analysis showed significantly higher levels of *scl1* transcription, particularly in M1, as well as in M28 and M41 strains, relative to M3-type strains analyzed.

Variation in the Mga coding sequence and autoregulated *mga* promoter has been reported to significantly affect Mga-dependent gene expression and virulence properties in GAS (Flores et al., 2013; Cao et al., 2014; Sanson et al., 2015). For example, a 12-bp deletion of a single repeat in the VNTR region of the *mga* promoter is present in M3 carrier strains and absent in the invasive M3 strains, causing a downregulation of Mga expression and Mga-regulated genes in the carrier strains (Flores et al., 2013). We identified an analogous 12-bp deletion in the VNTR of the *mga* promoter of the M1-type MGAS5005 and in M3 strains, MGAS335 and MGAS1313, but not in MGAS315, MGAS158, and MGAS10870. It is likely that the variation observed in the VNTR region impacts Mga expression but could not, alone, explain differences in *scl1* transcription patterns observed in this work. Consequently, qRT-PCR analyses showed *mga* and *emm* transcripts (*emm* is second downstream target of Mga within the Mga regulon) were expressed at similar levels in MGAS5005 and M3 strains, except for the MGAS335, indicating a mechanism of *scl1* downregulation in M3 strains, which is independent from the level of Mga transcription. The presence of *emm* transcripts and M3-protein products indicate that Mga is present and functional in the majority of M3 strains tested, and therefore the polymorphism we observed in the *mga* promoter did not affect Mga expression or activity of Mga-controlled *scl1.3* promoter. Altogether, our data hint at the insertion of the IS1548 in the downregulation of *scl1.3* expression, specifically in M3-type GAS, although additional studies are required to firmly establish whether the IS element insertion is responsible.

It is intriguing that *scl1* and *scl2* expression patterns were drastically different between M1 and M3-type GAS, both associated with invasive infections. The *scl1.3* transcript was decreased by 21-fold in MGAS315, and even further in the remaining M3 strains studied (additional 20- to 45-fold), as compared to the M1-type strain MGAS5005. The upregulation of *scl1* in M1-type GAS has previously been shown in invasive M1 isolates, including MGAS5005, as compared to pharyngeal M1 isolates (Sumby et al., 2006). Studies comparing wild-type and isogenic *scl1.1* mutants in M1-type GAS reported that Scl1.1 contributed to immune evasion by inhibiting neutrophil extracellular trap production and by protecting bacteria from the cathelicidin LL-37 (Döhrmann et al., 2014). We conclude the differences we observed in *scl1* expression are related to different pathogenicity requirements for Scl1 protein in M1- vs. M3-type strains. In contrast, Scl2 expression is highly upregulated in M3-type strains, as compared to invasive M1-type GAS. While the majority of M3-type strains contain in-frame *scl2.3* alleles, all 21 M1 strains we analyzed contained out-of-frame *scl2.1* alleles (unpublished data). Although, the Scl2.3 human ligands are not known, it may have an unidentified biological function, which is important for pathogenesis of M3, but not M1, strains. Hence, we are reporting striking differences in the expression and features of the Scl1 and Scl2 proteins that evolved in the invasive M1 and M3 strains.

### Scl1-mediated adhesion and biofilm formation *in vitro*

Scl1 mediates binding to human extracellular matrix components, cellular fibronectin and laminin, as well as biofilm formation (Caswell et al., 2010; Oliver-Kozup et al., 2011, 2013). Previous work identified that Scl1 binds specifically to the C-C′ loop of the type III-repeat EDA domain of cellular fibronectin (Oliver-Kozup et al., 2013). This represents a novel mechanism of fibronectin binding, which is distinct from the mechanism employed by other GAS fibronectin-binding proteins that bind to the N-terminal type I repeats of fibronectin (Yamaguchi et al., 2013). Here, we determined that rScl1.3FL binds to cellular fibronectin via the same C-C′ loop-dependent mechanism. It is important for our overall model to acknowledge that the EDA-cFn isoform is specifically produced during embryogenesis and during wound healing in adult tissue (Ffrench-Constant et al., 1989; Jarnagin et al., 1994; Singh et al., 2004), which indicates Scl1 evolved with a unique function for targeting wounded tissue, a presumed pathogen portal of entry. We also demonstrate significant binding of rScl1.3FL to laminin, a major basement membrane protein at the epidermal-dermal junction; none of those ECM ligands were bound by rScl2.3, consistent with previous knowledge that Scl1, but not Scl2, variants exhibit binding to cFn and Lm (Caswell et al., 2010). These studies show that full-length Scl1.3, if expressed on the M3-GAS cell surface, would bind ECM, contributing to tissue colonization.

The diminished Scl1.3 expression and poor biofilm formation, led us to hypothesize that lack of surface-expressed Scl1 in M3-type GAS reduces host ECM binding and stable microcolony formation in tissue, thus, shifting the balance toward invasive spread, augmented by other virulence factors expressed by these strains. Recombinant rScl1.3FL formed aggregates that were mediated by the V-to-V region interactions, which could represent a mechanism of biofilm and microcolony stabilization by Scl1 molecules on adjacent GAS cells. Two representative invasive M3 isolates, MGAS315 and MGAS10870, acquired biofilm formation on cFn and Lm when homologous complementation was performed with surface-attached Scl1.3FL. MGAS315 has been shown to contain a missense mutation in the *covS* gene, causing upregulation of CovRS-regulated virulence genes and enhanced virulence during subcutaneous infection of mice, compared to an isogenic strain containing the WT *covS* allele (Stetzner et al., 2015). Additionally, MGAS315 contains a mutation in the regulator of protein B allele, *ropB*, which produces a nonfunctional RopB variant, while MGAS10870 contains a wild-type *covR/S* and *ropB* alleles (Carroll et al., 2011), allowing us to demonstrate the effect of Scl1.3FL function in the presence of differing regulatory networks. Similarly, heterologous complementation with surface Scl1.3FL in a *scl1.41*-mutant of the non-invasive biofilm-capable strain M41-type MGAS6183, restored its biofilm capacity to the wild-type level. These results indicate that M3-derived Scl1.3FL variant has the full capacity to support biofilm formation to a similar degree as Scl1 from a divergent M-type. The robust biofilm observed *in vitro* on ECM coatings validates the concept that biofilm capacity combined with adherence to the surrounding host ECM would reinforce the formation of stable tissue microcolonies *in vivo*.

### *In vitro* skin equivalent model of wound colonization and microcolony formation

We observed inhibition of wound re-epithelization by GAS infection of wounded skin equivalents, as well as the thinning of the viable epidermal layer at sites distant from the infected wound. In addition to our study, others have reported changes in skin histopathology and wound healing, resulting from bacterial infections. An *in vivo* study has reported epidermal defects as a result of GAS infection in a humanized mouse model with human skin graft (Scaramuzzino et al., 2000). Previous study of M3-type GAS infection using a skin equivalent model showed that hyaluronic acid capsule interactions with CD44 receptor on keratinocytes induced intracellular signaling, resulting in cytoskeletal rearrangement and monolayer disruption (Cywes and Wessels, 2001). Infection of an *in vitro* skin model containing a burn wound with *Pseudomonas aeruginosa* caused a loss of the keratinocyte layer and basement membrane, while intact epidermis was observed in burned but uninfected tissue (Shepherd et al., 2009). Impairment of wound healing has also been demonstrated by staphylococcal infections. Infection of dermal wounds in rabbit ears with *Staphylococcus aureus* showed the formation of biofilm, production of a persistent, low grade inflammatory response, and significantly delayed wound healing (Gurjala et al., 2011). Similarly, delayed wound healing by both *S. aureus* and *S. epidermidis* biofilms was observed in a mouse model of cutaneous wounds (Schierle et al., 2009). The inhibition of wound healing we describe here is by and large consistent with reports by other laboratories generated using *in vivo* animal and *in vitro* human skin infection models.

Microcolonies have been identified within human streptococcal impetigo lesions (Akiyama et al., 2003) and in tonsils from patients with recurrent GAS pharyngeal tonsillitis (Roberts et al., 2012), and likely represent a superficial or persistent state of GAS colonization. However, streptococcal infections can result in invasive disease due to biofilm disruption and bacterial dissemination (Connolly et al., 2011a,b). We observed large rounded microcolonies formed in tissue during infection with M41 strain, while M3 GAS remained scattered throughout the tissue as single cells and chains. Microcolony formation has been previously observed with *S. aureus* infection in organotypic skin model (Popov et al., 2014). Moreover, wound infection in rabbit ears with *S. aureus* produced mature biofilms encased in exopolysaccharide, as revealed by concanavalin A staining (Gurjala et al., 2011). Similarly, we also demonstrated that microcolonies in M41-infected tissue were encapsulated in bacterial-associated glycocalyx. However, a lack of glycocalyx-encapsulated microcolonies was associated with infection by M3-type GAS.

These results support our hypothesis that biofilm-poor M3 strains are abolished in stable microcolony formation *in vivo*, in part due to a lack of the ECM-binding Scl1 protein and an overall lack of surface adhesins, although they likely express the FbaB protein, identified in M3 GAS to be involved in the adherence and invasion into epithelial and endothelial cells (Terao et al., 2001, 2002; Amelung et al., 2011).

### *In vivo* mouse model of skin infection

Recent study reported that a small proportion of non-invasive M3-type strains (~1.3%) were found to harbor the *scl1.3* “carrier allele,” which resulted from an in-frame deletion in the collagenous region, encompassing the null mutation, producing a shorter cell-attached Scl1.3 variant. This MGAS10870 strain containing the *scl1.3* carrier allele was attenuated following intramuscular infection (Flores et al., 2015). In this study, we utilized the M28- and M41-type strains, representing biofilm-rich producers, for subcutaneous inoculation. We observed that *scl1.28*- and *scl1.41*-inactivated isogenic mutants produced significantly larger skin lesions as compared to the wild-type parent strains. These results, again, support the hypothesis that lack of Scl1 surface adhesin destabilizes focused nidus of infection, resulting in a shift toward increased tissue spread. However, previous studies performed in a M1 GAS background, utilizing *scl1.1*-mutants for subcutaneous infection, reported smaller skin lesions in the mutant groups, which likely reflects a differing predominant function of Scl1.1 in M1-type GAS (Lukomski et al., 2000; Döhrmann et al., 2014). Investigations using intranasal and intraperitoneal mouse infection models of *Streptococcus pneumoniae* have shown that culture-grown bacteria disseminated to the ear and lungs, while biofilm-grown bacteria stably colonized the nasopharynx (Blanchette-Cain et al., 2013; Marks et al., 2013). A similar study on *Streptococcus pyogenes* showed that bacteria grown in biofilms have downregulated virulence genes and tend to colonize the nasal associated lymphoid tissue of mice, while culture-grown bacteria had significantly increased dissemination and were more virulent in a septicemia model (Marks et al., 2014). Previous studies reported that inactivation of some GAS genes resulted in increased skin pathology produced by the mutants compared to their wild-type organisms, and these genes often encoded surface proteins, including SpyCEP (Sumby et al., 2008), Mrp (Boyle et al., 1998), protein F1 (Nyberg et al., 2004), and Spy0128, encoding a major pilus subunit (Crotty Alexander et al., 2010). Similarly, the *covS* mutant of group A streptococcal M1T1 strain with upregulated SpeB-protease activity was hypervirulent and had reduced capacity to bind human epithelial cells and fibronectin, and also to form biofilm due to increased cleavage of surface proteins (Hollands et al., 2010). Altogether, the concept that expression of a surface adhesin, such as Scl1, involved in biofilm formation and host tissue attachment, is inversely related to strain invasiveness has gained support from several studies, including this study.

We show the invasive M1- and M3-type GAS evolved *scl1* and *scl2* alleles with opposite expression patterns, with *scl1* downregulated and *scl2* upregulated in M3 compared to M1 GAS. We show M3-type GAS, devoid of surface-expressed Scl1.3, lacked biofilm formation on ECM coatings and microcolony formation during infection of *in vitro* wounded skin equivalent. Complementation with surface Scl1.3FL restored biofilm capacity of M3-type GAS on ECM coatings. Mouse infection with the isogenic *scl1* mutants of biofilm-rich M28- and M41-type GAS produced larger lesions, supporting the role of Scl1 in a localized tissue infection. Lastly we developed a model for Scl1-mediated microcolony formation (Figure 10), whereby Scl1 expressed on the GAS surface strengthens host colonization by attachment to cFn and Lm expressed within wounded tissue, as well as biofilm formation via Scl1-Scl1 interactions, resulting in a local, stabilized microcolony. Conversely, when Scl1 is absent on the GAS cell surface, as is the case for M3-type GAS, bacteria lack stable anchoring in the surrounding host ECM, as well as structural strength within microcolony, promoting cells to disperse more freely.

## Author contributions

Conceived and designed the experiments: BB and SL; performed the experiments: BB, SC, AF, JM, DHM, CC, DB, DK, FS, and DM; analyzed the data: BB, SL, PL, TH, PC, and RB; wrote the paper: BB, SL. Read and approved the final manuscript: BB, SC, PL, TH, DHM, CC, DB, PC, DK, AF, JM, FS, DM, RB, and SL.

### Conflict of interest statement

The authors declare that the research was conducted in the absence of any commercial or financial relationships that could be construed as a potential conflict of interest.

## References

[B1] Akiyama H. Morizane S. Yamasaki O. Oono T. Iwatsuki K. (2003). Assessment of *Streptococcus pyogenes* microcolony formation in infected skin by confocal laser scanning microscopy. J. Dermatol. Sci. 32, 193–199. 10.1016/S0923-1811(03)00096-3 14507444

[B2] Almengor A. C. McIver K. S. (2004). Transcriptional activation of *sclA* by Mga requires a distal binding site in *Streptococcus pyogenes*. J. Bacteriol. 186, 7847–7857. 10.1128/JB.186.23.7847-7857.2004 15547255 PMC529090

[B3] Al Safadi R. Amor S. Hery-Arnaud G. Spellerberg B. Lanotte P. Mereghetti L.. (2010). Enhanced expression of *lmb* gene encoding laminin-binding protein in *Streptococcus agalactiae* strains harboring IS1548 in *scpB-lmb* intergenic region. PLoS ONE 5:e10794. 10.1371/journal.pone.0010794 20520730 PMC2875397

[B4] Amelung S. Nerlich A. Rohde M. Spellerberg B. Cole J. N. Nizet V.. (2011). The FbaB-type fibronectin-binding protein of *Streptococcus pyogenes* promotes specific invasion into endothelial cells. Cell. Microbiol. 13, 1200–1211. 10.1111/j.1462-5822.2011.01610.x 21615663 PMC4754676

[B5] Beres S. B. Carroll R. K. Shea P. R. Sitkiewicz I. Martinez-Gutierrez J. C. Low D. E.. (2010). Molecular complexity of successive bacterial epidemics deconvoluted by comparative pathogenomics. Proc. Natl. Acad. Sci. U.S.A. 107, 4371–4376. 10.1073/pnas.0911295107 20142485 PMC2840111

[B6] Beres S. B. Sylva G. L. Barbian K. D. Lei B. Hoff J. S. Mammarella N. D.. (2002). Genome sequence of a serotype M3 strain of group A *Streptococcus*: phage-encoded toxins, the high-virulence phenotype, and clone emergence. Proc. Natl. Acad. Sci. U.S.A. 99, 10078–10083. 10.1073/pnas.152298499 12122206 PMC126627

[B7] Beres S. B. Sylva G. L. Sturdevant D. E. Granville C. N. Liu M. Ricklefs S. M.. (2004). Genome-wide molecular dissection of serotype M3 group A *Streptococcus* strains causing two epidemics of invasive infections. Proc. Natl. Acad. Sci. U.S.A. 101, 11833–11838. 10.1073/pnas.0404163101 15282372 PMC511060

[B8] Blanchette-Cain K. Hinojosa C. A. Akula Suresh Babu R. Lizcano A. Gonzalez-Juarbe N. Munoz-Almagro C.. (2013). *Streptococcus pneumoniae* biofilm formation is strain dependent, multifactorial, and associated with reduced invasiveness and immunoreactivity during colonization. MBio 4, e00745–e00713. 10.1128/mBio.00745-13 24129258 PMC3812715

[B9] Boyle M. D. Raeder R. Flosdorff A. Podbielski A. (1998). Role of *emm* and *mrp* genes in the virulence of group A streptococcal isolate 64/14 in a mouse model of skin infection. J. Infect. Dis. 177, 991–997. 10.1086/515241 9534973

[B10] Cao T. N. Liu Z. Cao T. H. Pflughoeft K. J. Treviño J. Danger J. L.. (2014). Natural disruption of two regulatory networks in serotype M3 group A *Streptococcus* isolates contributes to the virulence factor profile of this hypervirulent serotype. Infect. Immun. 82, 1744–1754. 10.1128/IAI.01639-13 24516115 PMC3993421

[B11] Carapetis J. R. Steer A. C. Mulholland E. K. Weber M. (2005). The global burden of group A streptococcal diseases. Lancet Infect. Dis. 5, 685–694. 10.1016/S1473-3099(05)70267-X 16253886

[B12] Carroll R. K. Shelburne S. A. III Olsen R. J. Suber B. Sahasrabhojane P. Kumaraswami M.. (2011). Naturally occurring single amino acid replacements in a regulatory protein alter streptococcal gene expression and virulence in mice. J. Clin. Invest. 121, 1956–1968. 10.1172/JCI45169 21490401 PMC3083769

[B13] Caswell C. C. Lukomska E. Seo N. S. Höök M. Lukomski S. (2007). Scl1-dependent internalization of group A *Streptococcus* via direct interactions with the alpha2beta(1) integrin enhances pathogen survival and re-emergence. Mol. Microbiol. 64, 1319–1331. 10.1111/j.1365-2958.2007.05741.x 17542923

[B14] Caswell C. C. Oliver-Kozup H. Han R. Lukomska E. Lukomski S. (2010). Scl1, the multifunctional adhesin of group A *Streptococcus*, selectively binds cellular fibronectin and laminin, and mediates pathogen internalization by human cells. FEMS Microbiol. Lett. 303, 61–68. 10.1111/j.1574-6968.2009.01864.x 20002194 PMC2910189

[B15] Cleary P. P. Kaplan E. L. Handley J. P. Wlazlo A. Kim M. H. Hauser A. R.. (1992). Clonal basis for resurgence of serious *Streptococcus pyogenes* disease in the 1980s. Lancet 339, 518–521. 10.1016/0140-6736(92)90339-5 1346879

[B16] Cockerill F. R. III Macdonald K. L. Thompson R. L. Roberson F. Kohner P. C. Besser-Wiek J.. (1997). An outbreak of invasive group A streptococcal disease associated with high carriage rates of the invasive clone among school-aged children. JAMA 277, 38–43. 10.1001/jama.1997.03540250046030 8980208

[B17] Colman G. Tanna A. Efstratiou A. Gaworzewska E. T. (1993). The serotypes of *Streptococcus pyogenes* present in Britain during 1980-1990 and their association with disease. J. Med. Microbiol. 39, 165–178. 10.1099/00222615-39-3-165 8366514

[B18] Connolly K. L. Braden A. K. Holder R. C. Reid S. D. (2011a). Srv mediated dispersal of streptococcal biofilms through SpeB is observed in CovRS+ strains. PLoS ONE 6:e28640. 10.1371/journal.pone.0028640 22163320 PMC3233586

[B19] Connolly K. L. Roberts A. L. Holder R. C. Reid S. D. (2011b). Dispersal of Group A streptococcal biofilms by the cysteine protease SpeB leads to increased disease severity in a murine model. PLoS ONE 6:e18984. 10.1371/journal.pone.0018984 21547075 PMC3081844

[B20] Cramer T. Yamanishi Y. Clausen B. E. Förster I. Pawlinski R. Mackman N.. (2003). HIF-1alpha is essential for myeloid cell-mediated inflammation. Cell 112, 645–657. 10.1016/S0092-8674(03)00154-5 12628185 PMC4480774

[B21] Crotty Alexander L. E. Maisey H. C. Timmer A. M. Rooijakkers S. H. Gallo R. L. von Köckritz-Blickwede M.. (2010). M1T1 group A streptococcal pili promote epithelial colonization but diminish systemic virulence through neutrophil extracellular entrapment. J. Mol. Med. 88, 371–381. 10.1007/s00109-009-0566-9 19960175 PMC2843839

[B22] Cunningham M. W. (2000). Pathogenesis of group A streptococcal infections. Clin. Microbiol. Rev. 13, 470–511. 10.1128/CMR.13.3.470-511.2000 10885988 PMC88944

[B23] Cywes C. Wessels M. R. (2001). Group A *Streptococcus* tissue invasion by CD44-mediated cell signalling. Nature 414, 648–652. 10.1038/414648a 11740562

[B24] Davies H. D. Mcgeer A. Schwartz B. Green K. Cann D. Simor A. E.. (1996). Invasive group A streptococcal infections in Ontario, Canada. N. Engl. J. Med. 335, 547–554. 10.1056/NEJM199608223350803 8684408

[B25] Döhrmann S. Anik S. Olson J. Anderson E. L. Etesami N. No H.. (2014). Role for streptococcal collagen-like protein 1 in M1T1 group A *Streptococcus* resistance to neutrophil extracellular traps. Infect. Immun. 82, 4011–4020. 10.1128/IAI.01921-14 25024366 PMC4187857

[B26] DiPersio J. R. File T. M. Jr. Stevens D. L. Gardner W. G. Petropoulos G. Dinsa K. (1996). Spread of serious disease-producing M3 clones of Group A *Streptococcus* among family members and health care workers. Clin. Infect. Dis. 22, 490–495. 10.1093/clinids/22.3.490 8852968

[B27] Ffrench-Constant C. Van De Water L. Dvorak H. F. Hynes R. O. (1989). Reappearance of an embryonic pattern of fibronectin splicing during wound healing in the adult rat. J. Cell Biol. 109, 903–914. 10.1083/jcb.109.2.903 2760116 PMC2115730

[B28] Fields G. B. Noble R. L. (1990). Solid phase peptide synthesis utilizing 9-fluorenylmethoxycarbonyl amino acids. Int. J. Pept. Protein Res. 35, 161–214. 10.1111/j.1399-3011.1990.tb00939.x 2191922

[B29] Flores A. R. Jewell B. E. Versalovic E. M. Olsen R. J. Bachert B. A. Lukomski S.. (2015). Natural variant of collagen-like protein A in serotype M3 Group A *Streptococcus* increases adherence and decreases invasive potential. Infect. Immun. 83, 1122–1129. 10.1128/IAI.02860-14 25561712 PMC4333440

[B30] Flores A. R. Olsen R. J. Wunsche A. Kumaraswami M. Shelburne S. A. III. Carroll R. K.. (2013). Natural variation in the promoter of the gene encoding the Mga regulator alters host-pathogen interactions in Group A *Streptococcus* carrier strains. Infect. Immun. 81, 4128–4138. 10.1128/IAI.00405-13 23980109 PMC3811816

[B31] Gaworzewska E. Colman G. (1988). Changes in the pattern of infection caused by *Streptococcus pyogenes*. Epidemiol. Infect. 100, 257–269. 10.1017/S095026880006739X 3128449 PMC2249231

[B32] Granlund M. Oberg L. Sellin M. Norgren M. (1998). Identification of a novel insertion element, IS1548, in group B streptococci, predominantly in strains causing endocarditis. J. Infect. Dis. 177, 967–976. 10.1086/515233 9534970

[B33] Gurjala A. N. Geringer M. R. Seth A. K. Hong S. J. Smeltzer M. S. Galiano R. D.. (2011). Development of a novel, highly quantitative *in vivo* model for the study of biofilm-impaired cutaneous wound healing. Wound Repair Regen. 19, 400–410. 10.1111/j.1524-475X.2011.00690.x 21518094

[B34] Han R. Caswell C. C. Lukomska E. Keene D. R. Pawlowski M. Bujnicki J. M.. (2006a). Binding of the low-density lipoprotein by streptococcal collagen-like protein Scl1 of *Streptococcus pyogenes*. Mol. Microbiol. 61, 351–367. 10.1111/j.1365-2958.2006.05237.x 16856940

[B35] Han R. Zwiefka A. Caswell C. C. Xu Y. Keene D. R. Lukomska E.. (2006b). Assessment of prokaryotic collagen-like sequences derived from streptococcal Scl1 and Scl2 proteins as a source of recombinant GXY polymers. Appl. Microbiol. Biotechnol. 72, 109–115. 10.1007/s00253-006-0387-5 16552563

[B36] Hollands A. Pence M. A. Timmer A. M. Osvath S. R. Turnbull L. Whitchurch C. B.. (2010). Genetic switch to hypervirulence reduces colonization phenotypes of the globally disseminated Group A *Streptococcus* M1T1 clone. J. Infect. Dis. 202, 11–19. 10.1086/653124 20507231 PMC2880657

[B37] Hollm-Delgado M. G. Allard R. Pilon P. A. (2005). Invasive group A streptococcal infections, clinical manifestations and their predictors, Montreal, 1995-2001. Emerg. Infect. Dis. 11, 77–82. 10.3201/eid1101.030651 15705326 PMC3294357

[B38] Jarnagin W. R. Rockey D. C. Koteliansky V. E. Wang S. S. Bissell D. M. (1994). Expression of variant fibronectins in wound healing: cellular source and biological activity of the EIIIA segment in rat hepatic fibrogenesis. J. Cell Biol. 127, 2037–2048. 10.1083/jcb.127.6.2037 7806580 PMC2120289

[B39] Johnson D. R. Stevens D. L. Kaplan E. L. (1992). Epidemiologic analysis of group A streptococcal serotypes associated with severe systemic infections, rheumatic fever, or uncomplicated pharyngitis. J. Infect. Dis. 166, 374–382. 10.1093/infdis/166.2.374 1634809

[B40] Kaul R. Mcgeer A. Low D. E. Green K. Schwartz B. (1997). Population-based surveillance for group A streptococcal necrotizing fasciitis: clinical features, prognostic indicators, and microbiologic analysis of seventy-seven cases. Ontario Group A Streptococcal study. Am. J. Med. 103, 18–24. 10.1016/S0002-9343(97)00160-5 9236481

[B41] Lamagni T. L. Neal S. Keshishian C. Alhaddad N. George R. Duckworth G. (2008). Severe *Streptococcus pyogenes* infections, United Kingdom, 2003-2004. Emerg. Infect. Dis. 14, 202–209. 10.3201/eid1402.070888 18258111 PMC2600190

[B42] Lembke C. Podbielski A. Hidalgo-Grass C. Jonas L. Hanski E. Kreikemeyer B. (2006). Characterization of biofilm formation by clinically relevant serotypes of group A streptococci. Appl. Environ. Microbiol. 72, 2864–2875. 10.1128/AEM.72.4.2864-2875.2006 16597993 PMC1449035

[B43] Lukomski S. Montgomery C. A. Rurangirwa J. Geske R. S. Barrish J. P. Adams G. J.. (1999). Extracellular cysteine protease produced by *Streptococcus pyogenes* participates in the pathogenesis of invasive skin infection and dissemination in mice. Infect. Immun. 67, 1779–1788. 10085018 10.1128/iai.67.4.1779-1788.1999 PMC96528

[B44] Lukomski S. Nakashima K. Abdi I. Cipriano V. J. Ireland R. M. Reid S. D.. (2000). Identification and characterization of the *scl* gene encoding a Group A *Streptococcus* extracellular protein virulence factor with similarity to human collagen. Infect. Immun. 68, 6542–6553. 10.1128/IAI.68.12.6542-6553.2000 11083763 PMC97748

[B45] Lukomski S. Nakashima K. Abdi I. Cipriano V. J. Shelvin B. J. Graviss E. A.. (2001). Identification and characterization of a second extracellular collagen-like protein made by Group A *Streptococcus*: control of production at the level of translation. Infect. Immun. 69, 1729–1738. 10.1128/IAI.69.3.1729-1738.2001 11179350 PMC98079

[B46] Mahillon J. Leonard C. Chandler M. (1999). IS elements as constituents of bacterial genomes. Res. Microbiol. 150, 675–687. 10.1016/S0923-2508(99)00124-2 10673006

[B47] Marks L. R. Davidson B. A. Knight P. R. Hakansson A. P. (2013). Interkingdom signaling induces *Streptococcus pneumoniae* biofilm dispersion and transition from asymptomatic colonization to disease. MBio 4:e00438-13. 10.1128/mBio.00438-13 23882016 PMC3735180

[B48] Marks L. R. Mashburn-Warren L. Federle M. J. Hakansson A. P. (2014). *Streptococcus pyogenes* biofilm growth *in vitro* and *in vivo* and its role in colonization, virulence, and genetic exchange. J. Infect. Dis. 210, 25–34. 10.1093/infdis/jiu058 24465015 PMC4162002

[B49] McIver K. S. Scott J. R. (1997). Role of *mga* in growth phase regulation of virulence genes of the Group A *Streptococcus*. J. Bacteriol. 179, 5178–5187. 9260962 10.1128/jb.179.16.5178-5187.1997 PMC179378

[B50] Meisal R. Hoiby E. A. Caugant D. A. Musser J. M. (2010). Molecular characteristics of pharyngeal and invasive *emm3 Streptococcus pyogenes* strains from Norway, 1988-2003. Eur. J. Clin. Microbiol. Infect. Dis. 29, 31–43. 10.1007/s10096-009-0814-5 19806374

[B51] Musser J. M. Hauser A. R. Kim M. H. Schlievert P. M. Nelson K. Selander R. K. (1991). *Streptococcus pyogenes* causing toxic-shock-like syndrome and other invasive diseases: clonal diversity and pyrogenic exotoxin expression. Proc. Natl. Acad. Sci. U.S.A. 88, 2668–2672. 10.1073/pnas.88.7.2668 1672766 PMC51299

[B52] Nakagawa I. Kurokawa K. Yamashita A. Nakata M. Tomiyasu Y. Okahashi N.. (2003). Genome sequence of an M3 strain of *Streptococcus pyogenes* reveals a large-scale genomic rearrangement in invasive strains and new insights into phage evolution. Genome Res. 13, 1042–1055. 10.1101/gr.1096703 12799345 PMC403657

[B53] Nyberg P. Sakai T. Cho K. H. Caparon M. G. Fässler R. Björck L. (2004). Interactions with fibronectin attenuate the virulence of *Streptococcus pyogenes*. EMBO J. 23, 2166–2174. 10.1038/sj.emboj.7600214 15103329 PMC424380

[B54] Oliver-Kozup H. A. Elliott M. Bachert B. A. Martin K. H. Reid S. D. Schwegler-Berry D. E.. (2011). The streptococcal collagen-like protein-1 (Scl1) is a significant determinant for biofilm formation by Group A *Streptococcus*. BMC Microbiol. 11:262. 10.1186/1471-2180-11-262 22168784 PMC3268755

[B55] Oliver-Kozup H. Martin K. H. Schwegler-Berry D. Green B. J. Betts C. Shinde A. V.. (2013). The group A streptococcal collagen-like protein-1, Scl1, mediates biofilm formation by targeting the extra domain A-containing variant of cellular fibronectin expressed in wounded tissue. Mol. Microbiol. 87, 672–689. 10.1111/mmi.12125 23217101 PMC3556226

[B56] Olsen R. J. Laucirica D. R. Watkins M. E. Feske M. L. Garcia-Bustillos J. R. Vu C.. (2012). Polymorphisms in regulator of protease B (RopB) alter disease phenotype and strain virulence of serotype M3 Group A *Streptococcus*. J. Infect. Dis. 205, 1719–1729. 10.1093/infdis/jir825 22262791 PMC4447876

[B57] Påhlman L. I. Marx P. F. Mörgelin M. Lukomski S. Meijers J. C. Herwald H. (2007). Thrombin-activatable fibrinolysis inhibitor binds to *Streptococcus pyogenes* by interacting with collagen-like proteins A and B. J. Biol. Chem. 282, 24873–24881. 10.1074/jbc.M610015200 17553807

[B58] Popov L. Kovalski J. Grandi G. Bagnoli F. Amieva M. R. (2014). Three-dimensional Human Skin models to understand *Staphylococcus aureus* Skin colonization and infection. Front. Immunol. 5:41. 10.3389/fimmu.2014.00041 24567733 PMC3915142

[B59] Rasmussen M. Björck L. (2001). Unique regulation of SclB - a novel collagen-like surface protein of *Streptococcus pyogenes*. Mol. Microbiol. 40, 1427–1438. 10.1046/j.1365-2958.2001.02493.x 11442840

[B60] Rasmussen M. Eden A. Björck L. (2000). SclA, a novel collagen-like surface protein of *Streptococcus pyogenes*. Infect. Immun. 68, 6370–6377. 10.1128/IAI.68.11.6370-6377.2000 11035747 PMC97721

[B61] Roberts A. L. Connolly K. L. Kirse D. J. Evans A. K. Poehling K. A. Peters T. R.. (2012). Detection of Group A *Streptococcus* in tonsils from pediatric patients reveals high rate of asymptomatic streptococcal carriage. BMC Pediatr. 12:3. 10.1186/1471-2431-12-3 22230361 PMC3279307

[B62] Russo A. Scognamiglio P. L. Hong Enriquez R. P. Santambrogio C. Grandori R. Marasco D.. (2015). *In silico* generation of Peptides by Replica Exchange Monte Carlo: docking-based optimization of Maltose-Binding-Protein ligands. PLoS ONE 10:e0133571. 10.1371/journal.pone.0133571 26252476 PMC4529233

[B63] Sanson M. O'neill B. E. Kachroo P. Anderson J. R. Flores A. R. Valson C.. (2015). A naturally occurring single amino acid replacement in multiple gene regulator of Group A *Streptococcus* significantly increases virulence. Am. J. Pathol. 185, 462–471. 10.1016/j.ajpath.2014.10.018 25476528 PMC4305177

[B64] Scaramuzzino D. A. Mcniff J. M. Bessen D. E. (2000). Humanized *in vivo* model for streptococcal impetigo. Infect. Immun. 68, 2880–2887. 10.1128/IAI.68.5.2880-2887.2000 10768985 PMC97500

[B65] Schierle C. F. De La Garza M. Mustoe T. A. Galiano R. D. (2009). Staphylococcal biofilms impair wound healing by delaying reepithelialization in a murine cutaneous wound model. Wound Repair Regen. 17, 354–359. 10.1111/j.1524-475X.2009.00489.x 19660043

[B66] Sela S. Aviv A. Tovi A. Burstein I. Caparon M. G. Hanski E. (1993). Protein F: an adhesin of *Streptococcus pyogenes* binds fibronectin via two distinct domains. Mol. Microbiol. 10, 1049–1055. 10.1111/j.1365-2958.1993.tb00975.x 7934855

[B67] Sharkawy A. Low D. E. Saginur R. Gregson D. Schwartz B. Jessamine P.. (2002). Severe group a streptococcal soft-tissue infections in Ontario: 1992-1996. Clin. Infect. Dis. 34, 454–460. 10.1086/338466 11797171

[B68] Shea P. R. Beres S. B. Flores A. R. Ewbank A. L. Gonzalez-Lugo J. H. Martagon-Rosado A. J. (2011). Distinct signatures of diversifying selection revealed by genome analysis of respiratory tract and invasive bacterial populations. Proc. Natl. Acad. Sci. U.S.A. 108, 5039–5044. 10.1073/pnas.1016282108 21383167 PMC3064369

[B69] Shepherd J. Douglas I. Rimmer S. Swanson L. Macneil S. (2009). Development of three-dimensional tissue-engineered models of bacterial infected human skin wounds. Tiss. Eng. C Methods 15, 475–484. 10.1089/ten.tec.2008.0614 19292658

[B70] Shulman S. T. Tanz R. R. Kabat W. Kabat K. Cederlund E. Patel D. (2004). Group A streptococcal pharyngitis serotype surveillance in North America, 2000-2002. Clin. Infect. Dis. 39, 325–332. 10.1086/421949 15306998

[B71] Sims Sanyahumbi A. Colquhoun S. Wyber R. Carapetis J. R. (2016). Global disease burden of Group A Streptococcus, in Streptococcus pyogenes: Basic Biology to Clinical Manifestations, eds Ferretti J. J. Stevens D. L. Fischetti V.A. (Oklahoma City, OK: University of Oklahoma Health Sciences Center). 26866218

[B72] Singh P. Reimer C. L. Peters J. H. Stepp M. A. Hynes R. O. Van De Water L. (2004). The spatial and temporal expression patterns of integrin alpha9beta1 and one of its ligands, the EIIIA segment of fibronectin, in cutaneous wound healing. J. Invest. Dermatol. 123, 1176–1181. 10.1111/j.0022-202X.2004.23485.x 15610531

[B73] Soriano N. Vincent P. Moullec S. Meygret A. Lagente V. Kayal S. (2014). Closed genome sequence of non-invasive *Streptococcus pyogenes* M/*emm3* strain STAB902. Genome Announc 2:e00792-14. 10.1128/genomea.00792-14 25169855 PMC4148723

[B74] Squeglia F. Bachert B. De Simone A. Lukomski S. Berisio R. (2014). The crystal structure of the streptococcal collagen-like protein 2 globular domain from invasive M3-type Group A *Streptococcus* shows significant similarity to immunomodulatory HIV protein gp41. J. Biol. Chem. 289, 5122–5133. 10.1074/jbc.M113.523597 24356966 PMC3931070

[B75] Squeglia F. Bachert B. Romano M. Lukomski S. Berisio R. (2013). Crystallization and preliminary X-ray crystallographic analysis of the variable domain of Scl2.3, a streptococcal collagen-like protein from invasive M3-type *Streptococcus pyogenes*. Acta Crystallogr. Sect. F Struct. Biol. Cryst. Commun. 69, 1023–1025. 10.1107/S174430911302068X 23989154 PMC3758154

[B76] Stetzner Z. W. Li D. Feng W. Liu M. Liu G. Wiley J. (2015). Serotype M3 and M28 group A streptococci have distinct capacities to evade neutrophil and TNF-alpha responses and to invade soft tissues. PLoS ONE 10:e0129417 10.1371/journal.pone.0129417 26047469 PMC4457532

[B77] Stevens D. L. Kaplan E. L. (eds.). (2000). Streptococcal Infections: Clinical Aspects, Microbiology, and Molecular Pathogenesis. New York, NY: Oxford University Press.

[B78] Stevens D. L. Tanner M. H. Winship J. Swarts R. Ries K. M. Schlievert P. M.. (1989). Severe group A streptococcal infections associated with a toxic shock-like syndrome and scarlet fever toxin A. N. Engl. J. Med. 321, 1–7. 10.1056/NEJM198907063210101 2659990

[B79] Sumby P. Whitney A. R. Graviss E. A. DeLeo F. R. Musser J. M. (2006). Genome-wide analysis of group A streptococci reveals a mutation that modulates global phenotype and disease specificity. PLoS Pathog. 2:e5. 10.1371/journal.ppat.0020005 16446783 PMC1354197

[B80] Sumby P. Zhang S. Whitney A. R. Falugi F. Grandi G. Graviss E. A.. (2008). A chemokine-degrading extracellular protease made by Group A *Streptococcus* alters pathogenesis by enhancing evasion of the innate immune response. Infect. Immun. 76, 978–985. 10.1128/IAI.01354-07 18174342 PMC2258835

[B81] Terao Y. Kawabata S. Kunitomo E. Murakami J. Nakagawa I. Hamada S. (2001). Fba, a novel fibronectin-binding protein from *Streptococcus pyogenes*, promotes bacterial entry into epithelial cells, and the *fba* gene is positively transcribed under the Mga regulator. Mol. Microbiol. 42, 75–86. 10.1046/j.1365-2958.2001.02579.x 11679068

[B82] Terao Y. Kawabata S. Nakata M. Nakagawa I. Hamada S. (2002). Molecular characterization of a novel fibronectin-binding protein of *Streptococcus pyogenes* strains isolated from toxic shock-like syndrome patients. J. Biol. Chem. 277, 47428–47435. 10.1074/jbc.M209133200 12359713

[B83] Vanier G. S. (2013). Microwave-assisted solid-phase peptide synthesis based on the Fmoc protecting group strategy (CEM). Methods Mol. Biol. 1047, 235–249. 10.1007/978-1-62703-544-6_17 23943491

[B84] Virtaneva K. Porcella S. F. Graham M. R. Ireland R. M. Johnson C. A. Ricklefs S. M.. (2005). Longitudinal analysis of the Group A *Streptococcus* transcriptome in experimental pharyngitis in cynomolgus macaques. Proc. Natl. Acad. Sci. U.S.A. 102, 9014–9019. 10.1073/pnas.0503671102 15956184 PMC1150296

[B85] Whatmore A. M. (2001). *Streptococcus pyogenes sclB* encodes a putative hypervariable surface protein with a collagen-like repetitive structure. Microbiology 147, 419–429. 10.1099/00221287-147-2-419 11158359

[B86] Williamson M. P. (2013). Using chemical shift perturbation to characterise ligand binding. Prog. Nucl. Magn. Reson. Spectrosc. 73, 1–16. 10.1016/j.pnmrs.2013.02.001 23962882

[B87] Xu Y. Keene D. R. Bujnicki J. M. Höök M. Lukomski S. (2002). Streptococcal Scl1 and Scl2 proteins form collagen-like triple helices. J. Biol. Chem. 277, 27312–27318. 10.1074/jbc.M201163200 11976327

[B88] Yamaguchi M. Terao Y. Kawabata S. (2013). Pleiotropic virulence factor - *Streptococcus pyogenes* fibronectin-binding proteins. Cell. Microbiol. 15, 503–511. 10.1111/cmi.12083 23190012

