# Supplementary material for: Unique Footprint in the scl1.3 Locus Affects Adhesion and Biofilm Formation of the Invasive M3-Type Group A Streptococcus
Source: Front Cell Infect Microbiol. 2016 Aug 31;6:90. doi: 10.3389/fcimb.2016.00090 (PMC5005324; doi:10.3389/fcimb.2016.00090)
Supplement: Supplementary file 1 [file Table1.PDF]

**Table S1. Primers used in this study.**

| Primer name | Sequence (5'-3') | Use |
|---|---|---|
| **Analytical PCR** | | |
| IS1548F | GCCGTCTGCGTGCCCATTGCGTCTA | Detection of |
| Scl1 R | ACTAGATCTGAGATTATGGTGCTTTGATGTC | IS1548 |
| 232 Up | CTCCACAAAAGAGTGATCAGTC | Amplification |
| 232 Rev | TTAGTTGTTTTCTTTGCGTTT | of *scl1.3* |
| Scl2.3 F | AGGCATACAAGATCATGTCCTTGA | Amplification |
| Scl2.3 R | TTTGGTGTATGTGGTGCGGT | of *scl2.3* |
| Scl Up | CTTTCAATGGATGACGATACC | Amplification |
| Scl Rev | ACTTTCCATCAGTTAGGTAGC | of *scl2.3* |
| **Cloning** | | |
| Scl1.3 M3VF | GAGATGGCCGAGACTCCTATGACATCAAAGG | Cloning of |
| Scl1.3 M3VR | CAGCGTCTCAGCGCTCTTTGTTGCACCTTTTTCAA TCAG | *scl1.3V* region |
| 232 Up | CTCCACAAAAGAGTGATCAGTC | Cloning of |
| ME7 | TCAGTGAATTCTCTTTAGAGGATTAG | *scl1.3WT* |
| pJRS525F1 | GGGTTTTCCCAGTCACG | Repair of |
| ME6[a] | TCCAGCAGGACCTCGAGGT**GAA**CGC | *scl1.3* null |
| Scl1.3 M3VF | GAGATGGCCGAGACTCCTATGACATCAAAGG | Cloning of |
| Scl1.28WMR | GTCAAGCTTATTATTTTTCGAACTGCGGGTGGCTC CAAGGTTTTTCTGGAGCTGGAGTTACC | *scl1.3FL* |
| Scl1.3 M3VF ext | GAGATGGCCGAGACTCCTATGACATCAAAGGAGA GACAAG | Cloning of *scl1.3WT* |
| Scl1.3 truncR2 | GGTCTCAGCGCTACCTCGAGGTCCTGCTGGACCT TG | |
| Scl2 M3VF | GAGATGGCCGATGGTGAAGATGCCCAAAAAAG | Cloning of |
| Scl2.28_WMR | GTCAAGCTTATTATTTTTCGAACTGCGGGTGGCTC CAT | *scl2.3* |
| **qRT-PCR analysis** | | |
| tufA_F | CAACTCGTCACTATGCGCACAT | qRT-PCR of |
| tufA_R | GAGCGGCACCAGTGATCAT | *tufA* |
| Scl1_WMR_exp_F | TGCTGACAAAGAAGCTAACCAAAC | qRT-PCR of |
| Scl1_WMR_exp_R | GTGGTTGTTGGCTACAGGTGTCT | *scl1* |
| Scl2_WMR_exp_F | TCCTAAAACACCAGAGGTCC | qRT-PCR of |
| Scl2_WMR_exp_R2 | TGTGTGTGTCGTGAGCTGC | *scl2* |
| emm3_exp_F2 | AACAAATCTCAGACGCAAGCCGTC | qRT-PCR of |
| emm3_exp_R2 | TTCAAGCTCTTTGTTWAGTTTTTCAAG | *emm* |
| Mga_TMF | CAAGTCAACAGTGGAGAGAACTAAAATT | qRT-PCR of |
| Mga_TMR | ATGGAGATGTTGAGAGCTTTGCT | *mga* |
| Mga_F1 | ATCAGACAAAAACATTAAATTGCATG | Sequencing |
| Mga_R1 | TTGCATGTTAGTGAGACAAGTTTGC | of mga |

[a] Bolded GAA in primer ME6 indicates codon for glutamine in place of the original TAA stop codon.
