# Supplementary material for: Unique Footprint in the scl1.3 Locus Affects Adhesion and Biofilm Formation of the Invasive M3-Type Group A Streptococcus
Source: Front Cell Infect Microbiol. 2016 Aug 31;6:90. doi: 10.3389/fcimb.2016.00090 (PMC5005324; doi:10.3389/fcimb.2016.00090)
Supplement: Supplementary file 2 [file Table2.PDF]

Table S2. Variation in *scl2.3* gene among M3 strains.

Strain	No. of GXY repeats	No. of bp in the CL region	Amplicon size (bp)[a]	No. of CAAAA repeats	GTG in frame[b]
MGAS274	~125	~1125	~1206	8	yes
MGAS315	116	1044	1125	5	yes
MGAS335	~135	~1215	~1296	4	no
MGAS1313	~119	~1071	~1152	8	yes
AM3	~129	~1161	~1242	10	no
MGAS3375	17	153	234	5	yes
MGAS9517	113	1017	1098	8	yes
MGAS9622	111	999	1080	11	yes
MGAS9631	113	1017	1098	18	no
MGAS9716	110	990	1071	14	yes
MGAS9726	111	999	1080	11	yes
MGAS9739	119	1071	1152	5	yes
MGAS9760	113	1017	1098	11	yes
MGAS9780	106	954	1035	14	yes
MGAS9842	139	1251	1332	9	no
MGAS9852	103	927	1008	8	yes
MGAS10080	117	1053	1134	5	yes
MGAS10118	119	1071	1152	5	yes
MGAS10220	110	990	1071	12	no

[a] Amplicons were generated using primers Scl2.3 F/ R (**Table S1**)

[b] GTG is *scl2* start codon
