# Supplementary material for: Unique Footprint in the scl1.3 Locus Affects Adhesion and Biofilm Formation of the Invasive M3-Type Group A Streptococcus
Source: Front Cell Infect Microbiol. 2016 Aug 31;6:90. doi: 10.3389/fcimb.2016.00090 (PMC5005324; doi:10.3389/fcimb.2016.00090)
Supplement: Supplementary file 3 [file Table3.PDF]

Table S3. Mass spectrometry identification of rScl and native Scl proteins[a].

Sample	No. of peptides >95% confident	Peptide	Confidence	Sequence coverage	Confident ID
rScl1.3V	4	EENSQEELK	99%	40%	yes
		EENSQEELKNFTEER	99%		
		LKEILDLIEK	99%		
		WYGTYFKEENSQEELK	99%		
rScl2.3	2	GIQDHVLDGQDGDR	99%	18%	yes
		EELLSALIDGTSR	98.13%		
**MGAS315 WT**					
Scl2.3 in Sup	12	DVTPAPQNPSN	99%	17%	yes
		DVTPAPQNPSNR	99%		
		EELLSALIDGTSR	99%		
		GEAGPAGPR	99%		
		GIQDHVLDGQDGDR	99%		
		GIQDHVLDGQDGDRGEAGPAGPR	99%		
		GLNKPQTQGGNQL	99%		
		GLNKPQTQGGNQLAK	99%		
		NKPQTQGGNQLAK	99%		
		REELLSALIDGTSR	99%		
		TPEVPQKPDTAPHTPK	99%		
		TPQIPGQSK	99%		
Scl2.3 in CW	7	DVTPAPQNPSNR	99%	15%	yes
		EELLSALIDGTSR	99%		
		GIQDHVLDGQDGDR	99%		
		GIQDHVLDGQDGDRGEAGPAGPR	99%		
		GLNKPQTQGGNQLAK	99%		
		REELLSALIDGTSR	99%		
		TPEVPQKPDTAPHTPK	99%		
**Scl1.3FL- complemented GAS**					
315WT :: *scl1.3FL*	5	EENSQEELKNFTEER	99%	17%	yes
		EILDLIEK	99%		
		GDKGETGLAGPVGPAGK	99%		
		GETGLAGPVGPAGK	99%		
		LKEILDLIEK	99%		
10870Δ*scl1* :: *scl1.3FL*	4	EILDLIEK	99%	11%	yes
		GDKGETGLAGPVGPAGK	99%		
		GETGLAGPVGPAGK	99%		
		LKEILDLIEK	99%		
M41Δ*scl1* :: *scl1.3FL*	3	EENSQEELKNFTEER	99%	13%	yes
		GDKGETGLAGPVGPAGK	99%		
		GETGLAGPVGPAGK	99%		

[a] Protein identification is confident by the presence of at least two distinct peptides with >95% confidence each. Sup, supernatant protein fractions; CW, cell-wall association protein fractions.
