# Supplementary material for: Unique Footprint in the scl1.3 Locus Affects Adhesion and Biofilm Formation of the Invasive M3-Type Group A Streptococcus
Source: Front Cell Infect Microbiol. 2016 Aug 31;6:90. doi: 10.3389/fcimb.2016.00090 (PMC5005324; doi:10.3389/fcimb.2016.00090)
Supplement: Supplementary file 4 [file Table4.PDF]

Table S4. Summary table of fold-changes in *scl1* and *scl2* expression[a].

Strain	Gene	Fold change	+SE	- SE	P value	Significance
M1	*scl1*	21.05195084	-2.33583	-2.62734	0.001184755	**
	scl2	-23.6920597	-1.97551	-1.82347	0.01549836	*
M28	*scl1*	6.237987683	-1.1098	-1.34997	0.01395444	*
	scl2	-7.25032516	0.960352	1.106978	0.034822349	*
M41	*scl1*	7.787441864	-0.82018	-0.91673	0.004897482	**
	scl2	-3.22615419	0.376259	0.425935	0.141911016	N.S.
**M3 MGAS:**						
10870	*scl1*	-25.2160816	1.366976	1.445328	5.48581E-06	***
	scl2	-1.84753589	-0.01174	-0.01166	0.077646106	N.S.
158	*scl1*	-45.8489636	-0.86051	-0.84465	0.000121468	***
	scl2	1.077129939	0.059307	0.056212	0.753558444	N.S.
335	*scl1*	-42.9309881	-4.03211	-3.68593	5.18378E-05	***
	scl2	-2.44408041	-1.65928	-0.98832	0.283398851	N.S.
1313	*scl1*	-43.929358	0.938527	0.959016	5.45812E-06	***
	scl2	-3.89390614	-2.38312	-1.47835	0.117229672	N.S.

[a]Fold-changes in *scl1* and *scl2* expression relative to M3 MGAS315 are shown for each strain. Data is based on qRT-PCR analysis of RNA isolated during exponential growth phase and expression is normalized to the expression of *tufA* gene. +SE, positive standard error; -SE, negative standard error. Significance is calculated based on three independent experiments, each performed in triplicate wells. *P<0.05, **P<0.01, ***P<0.001; student's *t*-test.
