# Supplementary figures and images for: Unique Footprint in the scl1.3 Locus Affects Adhesion and Biofilm Formation of the Invasive M3-Type Group A Streptococcus
Source: Front Cell Infect Microbiol. 2016 Aug 31;6:90. doi: 10.3389/fcimb.2016.00090 (PMC5005324; doi:10.3389/fcimb.2016.00090)

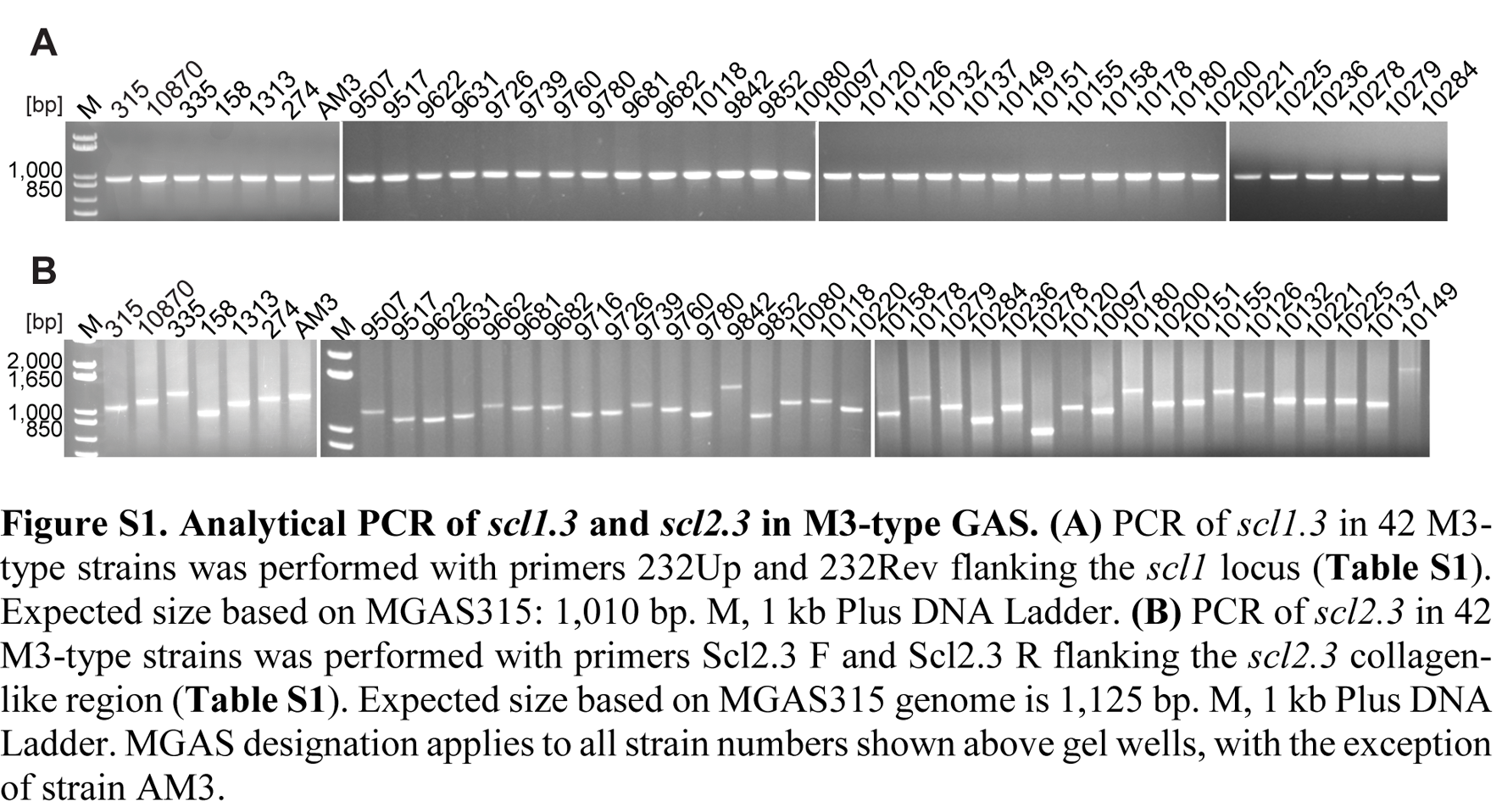

Supplement: Supplementary file 6 [file Image1.TIF]

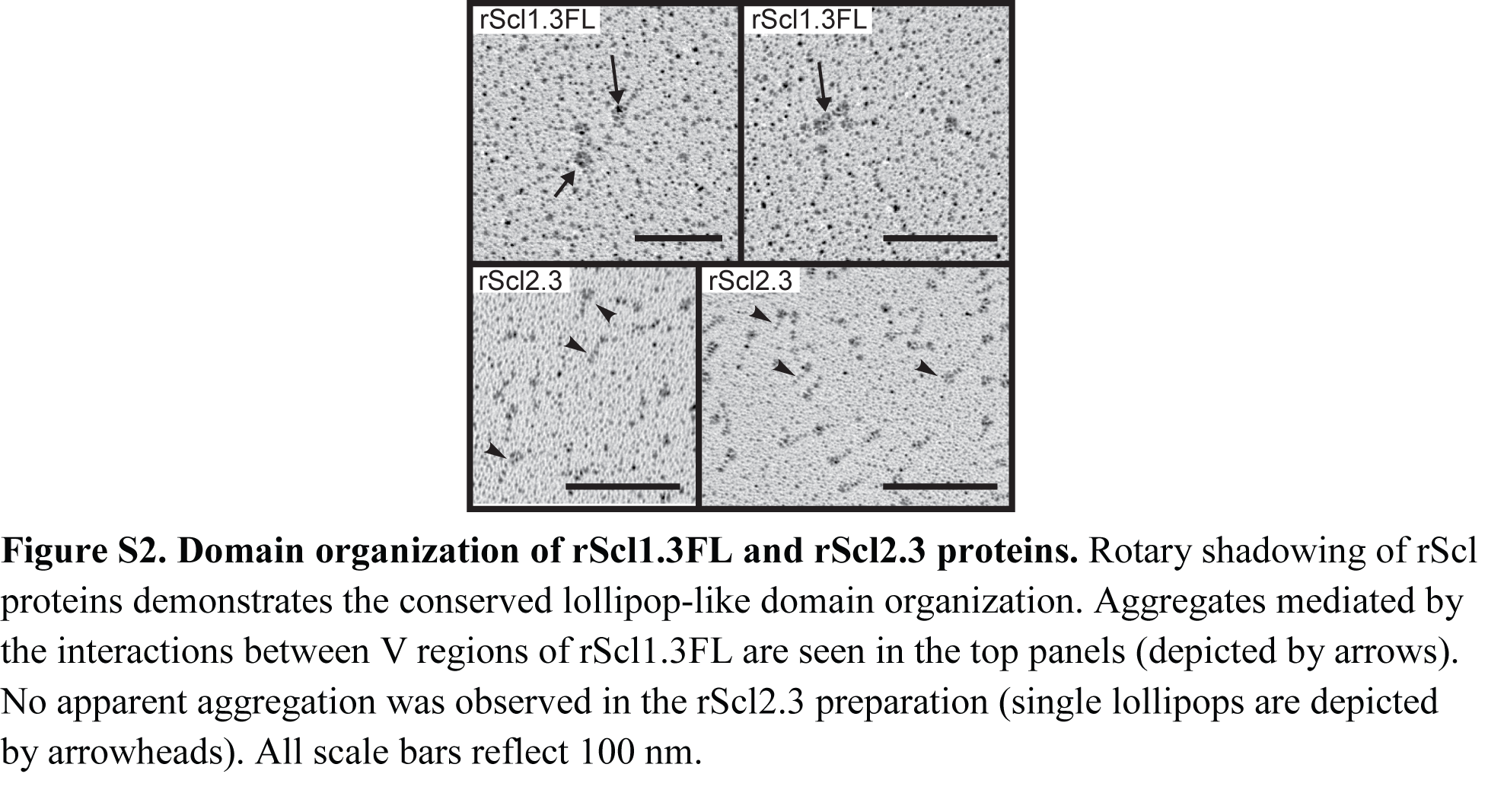

Supplement: Supplementary file 7 [file Image2.TIF]

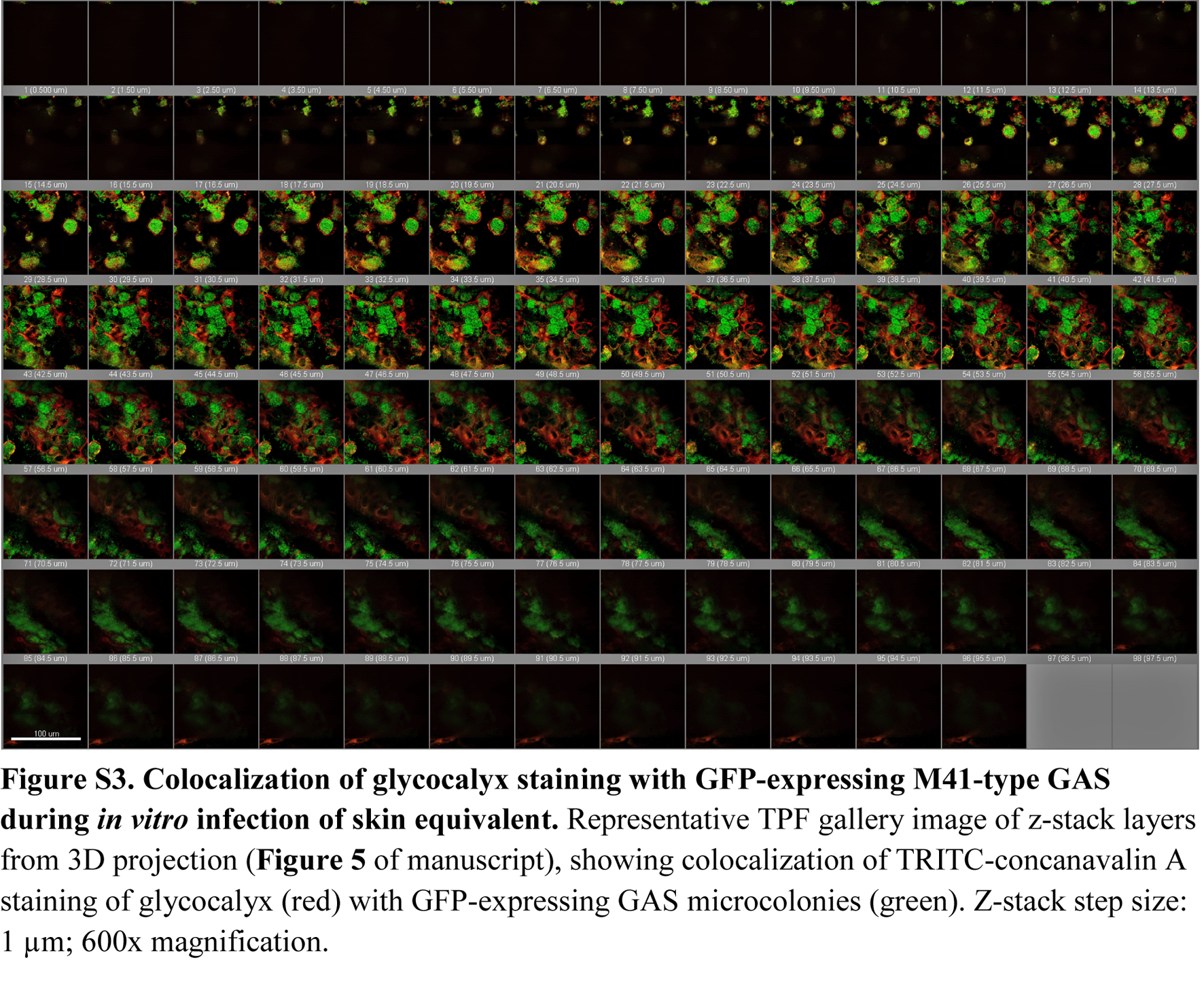

Supplement: Supplementary file 8 [file Image3.TIF]

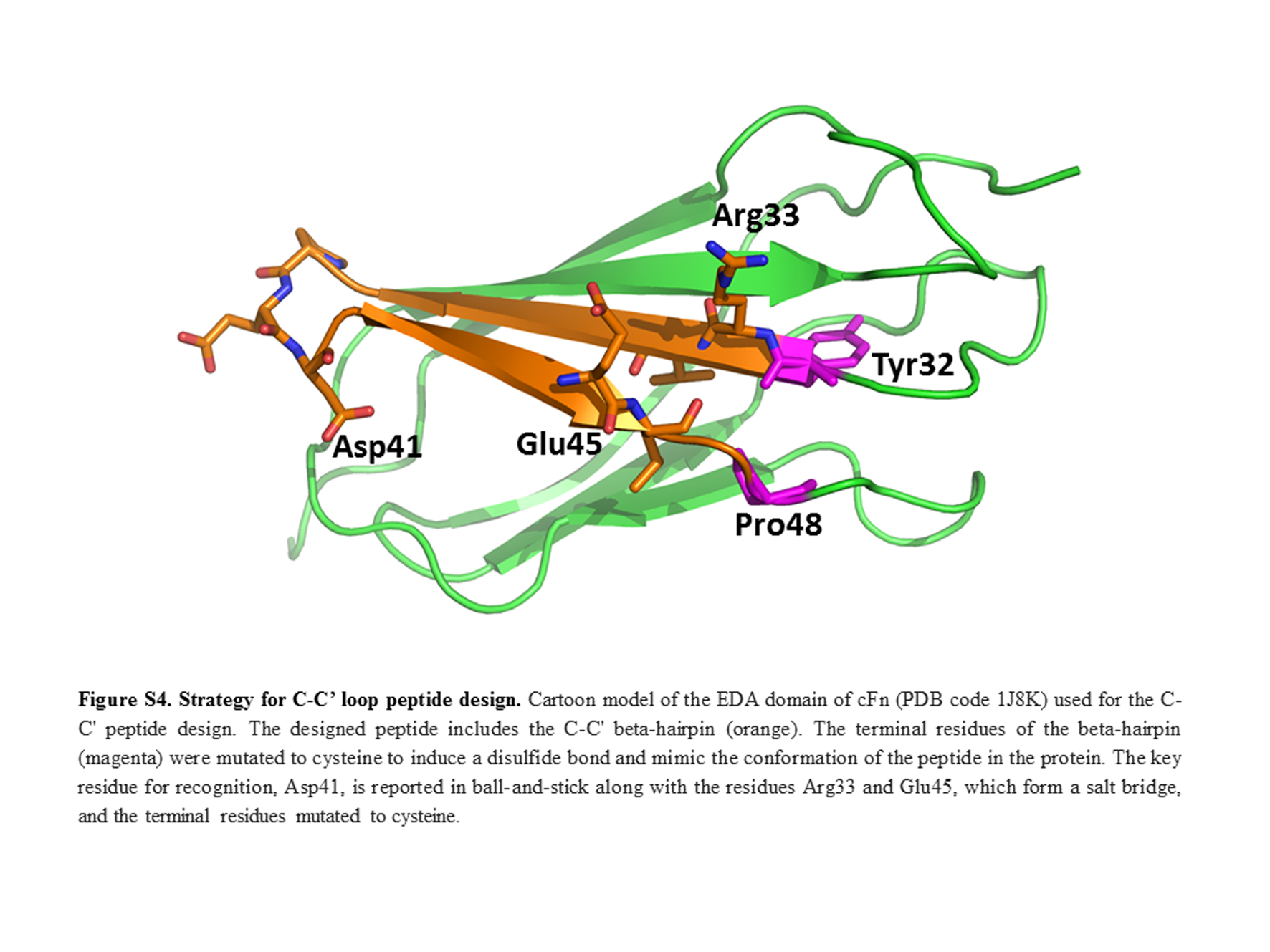

Supplement: Supplementary file 9 [file Image4.TIF]
